# Elevated Fcy receptor expression augments pro-inflammatory macrophage phagocytosis in systemic sclerosis and associated rheumatic diseases

**DOI:** 10.1093/rheumatology/keae688

**Published:** 2024-12-13

**Authors:** Amela Hukara, Gino A Bonazza, Tracy Tabib, Raphael Micheroli, Suzana Jordan, Kristina Bürki, Michal Rudnik, Adrian Ciurea, Oliver Distler, Robert Lafyatis, Przemysław Błyszczuk, Gabriela Kania

**Affiliations:** Center of Experimental Rheumatology, Department of Rheumatology, University Hospital Zurich, University of Zurich, Zurich, Switzerland; Center of Experimental Rheumatology, Department of Rheumatology, University Hospital Zurich, University of Zurich, Zurich, Switzerland; Division of Rheumatology and Clinical Immunology, Department of Medicine, University of Pittsburgh, Pittsburgh, Pennsylvania, USA; Center of Experimental Rheumatology, Department of Rheumatology, University Hospital Zurich, University of Zurich, Zurich, Switzerland; Center of Experimental Rheumatology, Department of Rheumatology, University Hospital Zurich, University of Zurich, Zurich, Switzerland; Center of Experimental Rheumatology, Department of Rheumatology, University Hospital Zurich, University of Zurich, Zurich, Switzerland; Center of Experimental Rheumatology, Department of Rheumatology, University Hospital Zurich, University of Zurich, Zurich, Switzerland; Center of Experimental Rheumatology, Department of Rheumatology, University Hospital Zurich, University of Zurich, Zurich, Switzerland; Center of Experimental Rheumatology, Department of Rheumatology, University Hospital Zurich, University of Zurich, Zurich, Switzerland; Division of Rheumatology and Clinical Immunology, Department of Medicine, University of Pittsburgh, Pittsburgh, Pennsylvania, USA; Center of Experimental Rheumatology, Department of Rheumatology, University Hospital Zurich, University of Zurich, Zurich, Switzerland; Center of Experimental Rheumatology, Department of Rheumatology, University Hospital Zurich, University of Zurich, Zurich, Switzerland

**Keywords:** macrophage activation, SSc, rheumatic autoimmune inflammatory diseases, single-cell transcriptomics, phagocytosis, fc gamma receptors, inflammatory pathways

## Abstract

**Objectives:**

To investigate the pro-phagocytic phenotype of macrophages in SSc and other rheumatic diseases by examining their activation, signalling pathways and treatment responses, with the goal of uncovering mechanisms that drive enhanced phagocytosis.

**Methods:**

Single-cell RNA sequencing (scRNA-seq) datasets (GSE138669/GSE212109) from skin and lung macrophages of healthy controls (HC) and SSc patients were analysed. Human monocyte-derived macrophages (hMDMs) were differentiated from CD14^+^ monocytes from HC, SSc, RA, PsA, and axSpA patients. In selected experiments, hMDMs were pretreated with 0.1 μM nintedanib. Phagocytic activity was quantified using pHrodo bioparticles and flow cytometry. Macrophage surface markers were evaluated by flow cytometry, NF-κB signalling by Western blot and gene expression by RT-qPCR.

**Results:**

Analysis of scRNA-seq datasets revealed a pro-phagocytic signature in SSc-affected organs. SSc macrophages, particularly the *FCGR3A*^hi^ cluster in skin, exhibited elevated expression of *FCGR* genes and enriched FcγR-mediated phagocytosis pathways, accompanied by pro-inflammatory markers. This phenotype extended to *FCN1*^hi^ lung macrophages in SSc patients with interstitial lung disease, indicating a systemic pro-inflammatory and phagocytic profile. hMDMs from SSc, RA and PsA patients demonstrated enhanced phagocytic activity *in vitro*. Elevated FcγRI and FcγRII levels were identified as key drivers of increased phagocytic activity and subsequent IL-6-driven inflammation. Nintedanib showed reduction in FcγRI expression, suggesting its potential therapeutic benefit in attenuating the phagocytic process.

**Conclusion:**

This study highlights FcγR-expressing macrophages as drivers of phagocytosis and inflammatory responses in SSc. Dysregulated activation of these macrophages could lead to persistent inflammation and fibrosis in rheumatic diseases, highlighting new potential therapeutic approaches.

## Graphical abstract

**Figure:**
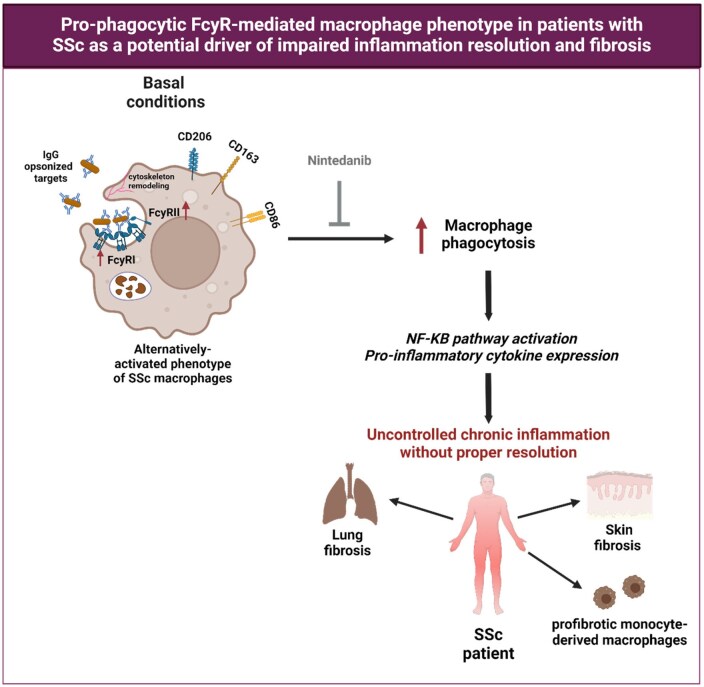

Rheumatology key messages

- A distinct pro-phagocytic and pro-inflammatory signature is identified in macrophages from SSc-affected organs, with elevated expression of Fcγ receptors.
- Nintedanib selectively reduces FcγRI expression on macrophages, indicating potential to attenuate excessive phagocytosis and inflammation in SSc.
- Enhanced phagocytic activity is not unique to SSc but also present in macrophages from RA and PsA patients.

## Introduction

SSc is a rheumatic autoimmune inflammatory disease (RAID), with its main hallmarks being microvascular abnormalities, multi-organ inflammation and fibrosis of the skin and internal organs [1]. Fibrosis is considered the outcome of the pathophysiological process of the disease, and current treatments can only slow disease progression of certain organ manifestations with no disease-modifying treatments available [2, 3]. SSc patients fall into two primary subtypes based on skin involvement. Limited cutaneous SSc (lcSSc) primarily affects fingers, distal extremities and sometimes the face. In contrast, diffuse cutaneous SSc (dcSSc) extends to these areas as well as the proximal extremities and trunk, with a higher risk of severe internal organ complications like interstitial lung disease (ILD) [1].

The exact aetiology of the disease remains unknown. However, research suggests that dysregulated inflammatory processes may result in sustained and uncontrolled inflammation, ultimately leading to fibrosis [4, 5]. Macrophages, crucial components of the innate immune system, are among the first responders to tissue damage and pathogens. [6, 7]. They play a central role in phagocytosis, the process by which cells engulf and digest foreign particles and debris. This process is initiated by the binding of opsonic receptors, like Fcγ receptors (FcγRs), to IgG antibodies [8]. The crosslinking of FcγRs triggers a phosphorylation cascade, leading to actin cytoskeleton reorganization [9] and the induction of pro-inflammatory responses [10]. Consequently, when unregulated, this can result in excessive inflammation. A recent study reported that phagocytosis of SARS-CoV-2-infected cells by monocyte-derived macrophages leads to the secretion of pro-inflammatory IL-6 and TNF, but also to an activation of plasmacytoid dendritic cells, followed by enhanced IFN-α and TNF levels [11]. This research highlights how macrophage phagocytosis triggers a pro-inflammatory response and links it to the activation of a type I IFN response. The presence of a type I IFN signature in SSc underscores its importance in understanding the disease's pathophysiology [12].

While macrophages in SSc are known to exhibit an alternatively activated profile, the specific role of FcγRs and phagocytosis in SSc remains underexplored [13, 14]. Alterations in FcγR expression and function have been noted in other autoimmune diseases such as SLE [15] and RA [16].

Our investigation aims to determine whether macrophages from SSc and other RAIDs display a pro-phagocytic phenotype and to assess the implications of this phenotype in promoting aberrant inflammation. Our findings are expected to highlight the critical role of FcγRs in driving heightened phagocytic activity and inflammatory pathways in these disorders, supporting the hypothesis that FcγR-dependent macrophage phagocytosis contributes significantly to the pathogenesis of autoimmune inflammatory diseases.

## Methods

### RAID patients and healthy controls

The human subject research study was conducted with the approval of the Cantonal ethics committee Zurich (KEK-ZH-NR. 515, PB 2016–02014, KEK-Nr.2018–01873). Blood samples from healthy controls (HC) and patients with RAIDs [SSc, RA, PsA and axSpA patients] were collected at the Department of Rheumatology of the University Hospital of Zurich (Switzerland). Informed consent was obtained from all participants.

The demographic and clinical characteristics of the different patient groups used for correlation with phagocytosis data are shown in Supplementary Tables S1 and S2, available at *Rheumatology* online.

### Statistics

Statistical analysis of experimental data was performed using GraphPad Prism 9 software. Data distribution was evaluated using the Shapiro-Wilk test. Normally distributed groups were analysed by unpaired two-tailed parametric *t* test or a paired *t* test and data are presented as mean (s.d.). Non-normally distributed data were analysed with unpaired non-parametric Mann–Whitney *U* test and data were displayed as median ± interquartile range. When comparing more than two normally distributed groups, one-way ANOVA test with Tukey’s multiple comparison was used. For comparison of more than two non-normally distributed groups, Kruskal-Wallis test with multiple comparisons was performed. More than three groups were compared with a two-way ANOVA with Bonferroni’s multiple comparison tests. One sample *t* test was performed for two groups when the data were compared to the control group, with a mean of 1. Correlations between clinical parameters and phagocytosis were assessed using Pearson’s correlation analysis. *P*-values < 0.05 were considered as statistically significant. The number of biological replicates (*n*) are given in each figure legend.

More information on material and methods is provided in the online Supplementary Materials (Supplementary Data S1, available at *Rheumatology* online).

## Results

### Skin and lung macrophages from SSc patients display a pro-phagocytic signature

**Figure 1.**
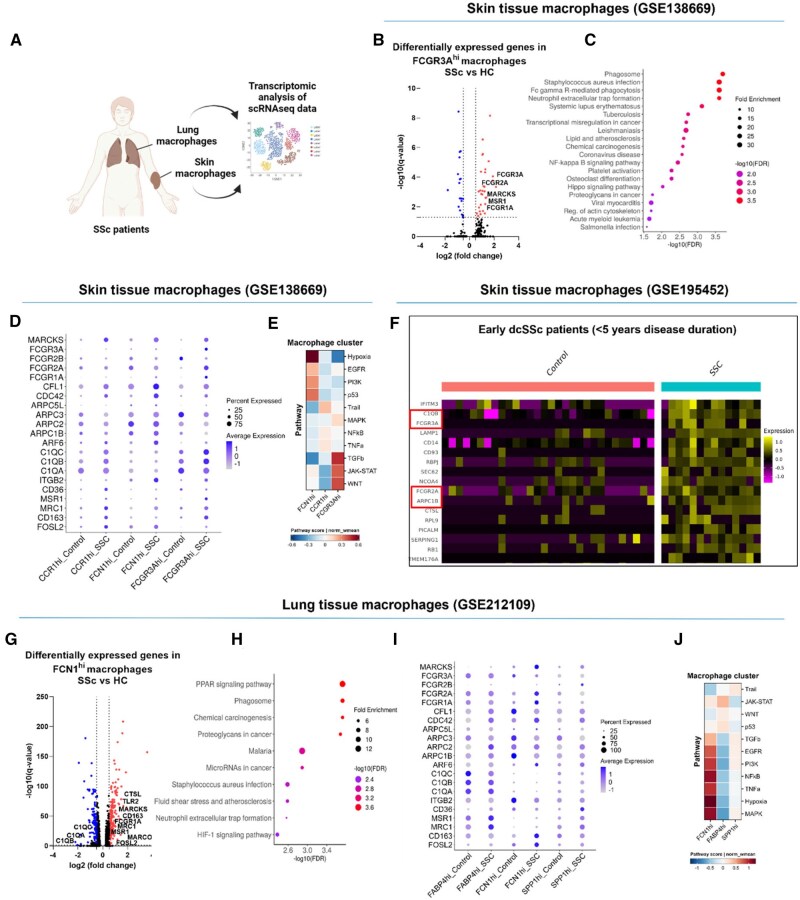
Skin and lung tissue macrophages from SSc patients display a pro-phagocytic signature. (A) Schematic representation of skin and lung tissue transcriptomic analysis. (B) Log_10_-adjusted *P* values (q-value) vs log2 fold changes of DEG comparing dcSSc patients vs HC in *FCGR3A*^hi^ skin macrophages. (C) Upregulated pathways comparing dcSSc patients vs HC in *FCGR3A*^hi^ skin macrophages. (D) Dot plot of a selection of macrophage polarization or phagocytosis-associated genes is shown comparing HC vs dcSSc patients in *CCR1*^hi^, *FCN1*^hi^ and *FCGR3A*^hi^ skin macrophages. (E) Heatmap of pathway scores is shown comparing the different *CCR1*^hi^, *FCN1*^hi^ and *FCGR3A*^hi^ skin macrophages from dcSSc patients. (F) Heatmap of DEG from the pseudobulk analysis comparing early dcSSc patients (disease duration <5 years) and HC in the total skin macrophage population (only DEG with log2FC ≥ 0.5 and *P* value < 0.05 are shown). (G) Log_10_-adjusted *P* values (*q*-value) versus log2 fold changes of DEG comparing SSc-ILD patients vs HC in *FCN1*^hi^ lung macrophages. (H) Upregulated pathways comparing SSc-ILD patients vs HC in *FCN1*^hi^ lung macrophages (FDR). (I) Dot plot of a selection of macrophage polarization or phagocytosis-associated genes is visualized comparing HC vs SSc-ILD patients in *FABP4*^hi^, *FCN1*^hi^ and *SPP1*^hi^ lung macrophages. (J), Heatmap of pathway scores is shown comparing the different *FABP4*^hi^, *FCN1*^hi^ and *SPP1*^hi^ lung macrophages from SSc-ILD patients. Illustration created with BioRender.com

To explore the pro-phagocytic characteristics of tissue macrophages in SSc, we analysed macrophage populations from 10 HC and 12 dcSSc patients using the skin single-cell RNA sequencing (scRNA-seq) dataset (GSE138669) (Fig. 1A) [17]. We identified three main macrophage subclusters—*FCN1*^hi^, *CCR1*^hi^ and *FCGR3A*^hi^ (Supplementary Fig. S1A, available at *Rheumatology* online)—based on the top differentially expressed genes (DEGs) (Supplementary Table S3a, available at *Rheumatology* online) [17]. Transcriptomic analysis revealed that *FCGR3A*^hi^ skin macrophages were distinguished by upregulated FCGR family genes (*FCGR1A*, *FCGR2A*, *FCGR3A*) (Fig.1B, Supplementary Table S3B, available at *Rheumatology* online) and enriched FcyR-mediated phagocytosis pathways (Fig. 1C), identifying them as the primary pro-phagocytic cluster in dcSSc skin. These macrophages also exhibited a pro-inflammatory profile, marked by upregulated NF-κB signalling (Fig 1C) and an alternatively activated phenotype characterized by *MSR1* expression (Fig 1B). Additionally, genes involved in phagocytosis (e.g. *MARCKS*) and macrophage polarization (e.g. *MSR1, CD163*) were elevated in *FCGR3A*^hi^ and *CCR1*^hi^ macrophages (Fig. 1D, Supplementary Fig. S2A and Tables S3B–D, available at *Rheumatology* online), with *FCGR3A*^hi^ showing a pronounced fibrotic signature through enhanced TGFβ, JAK-STAT and WNT pathway scores (Fig. 1E).

Further analysis of the population-scale massively parallel single-cell RNA sequencing (MARS-seq) dataset, which includes skin samples from 97 SSc patients (both lcSSc and dcSSc) and 56 HC [18], revealed only one significant DEG with log2FC ≥ 0.5 (*IFITM3*) in the overall skin macrophage population (Supplementary Table S4, available at *Rheumatology* online). Stratifying patients into the different SSc subtypes, mainly 32 lcSSc and 39 dcSSc, revealed no significant upregulated DEG in lcSSc (Supplementary Table S4, available at *Rheumatology* online). However, we identified seven upregulated DEGs in dcSSc patients (*IFITM3, IFI27, C1QB, FCGR3A, ARPC1B, LAPTM5, FYB1*) (Supplementary Table S4, available at *Rheumatology* online). Notably, early dcSSc patients (disease duration <5 years) exhibited 17 enriched DEGs (*C1QB, FCGR3A, FCGR2A, ARPC1B, CTSL*) (Fig. 1F), while late dcSSc patients (disease duration >5 years) showed 9 upregulated DEGs (*ARPC1B, FCGR3A*) (Supplementary Table S4, available at *Rheumatology* online), indicating a more pronounced pro-phagocytic signature and suggesting its significance in the early stages of the disease, particularly dcSSc.

After identifying a pro-phagocytic signature in SSc skin macrophages, we assessed this phenotype in lung macrophages from SSc-ILD patients. Analysis of lung macrophages from 5 SSc-ILD patients and 6 HC (GSE212109) [19] revealed key subclusters—*SPP1*^hi^, *FCN1*^hi^ and *FAPB4*^hi^ (Supplementary Fig. S1B, available at *Rheumatology* online)—based on a selection of the most upregulated DEGs (Supplementary Table S5A, available at *Rheumatology* online). SSC-ILD *FCN1*^hi^ macrophages were associated with upregulated FcγR-mediated phagocytosis (*FCGR1A, CTSL, MARCKS*) and alternatively activated macrophage markers (*MSR1, MRC1*) (Fig. 1G, Supplementary Table S5b, available at *Rheumatology* online). Complement component genes (*C1QA, C1QB, C1QC*) were downregulated in SSc-ILD *FCN1*^hi^ macrophages (Fig. 1G, Supplementary Table S5B, available at *Rheumatology* online) and gene ontology analysis revealed the phagosome pathway among the most enriched pathways (Fig. 1H), pointing towards a pro-phagocytic macrophage signature. Furthermore, genes involved in FcγR-mediated phagocytosis (such as *MARCKS*, *CDC42*) and macrophage polarization phenotypes (including *MSR1*, *MRC1*) were enriched in *FCN1^h^* and *SPP1*^hi^ lung macrophages from SSc-ILD patients (Fig. 1I, Supplementary Fig. S2B and Tables S5B–D, available at *Rheumatology* online). In contrast to the fibrotic signature of *FCGR3A*^hi^ skin macrophages, the *FCN1*^hi^ lung macrophages showed a stronger pro-inflammatory signature with enhanced NF-κB, TNFα, hypoxia, and MAPK pathway scores when compared with the *FABP4*^hi^ and the *SPP1*^hi^ macrophage clusters (Fig. 1J). Overall, these findings reveal a pro-phagocytic and alternatively activated phenotype in SSc tissue macrophages.

### Human monocyte-derived macrophages from SSc patients display increased pro-phagocytic activity

**Figure 2.**
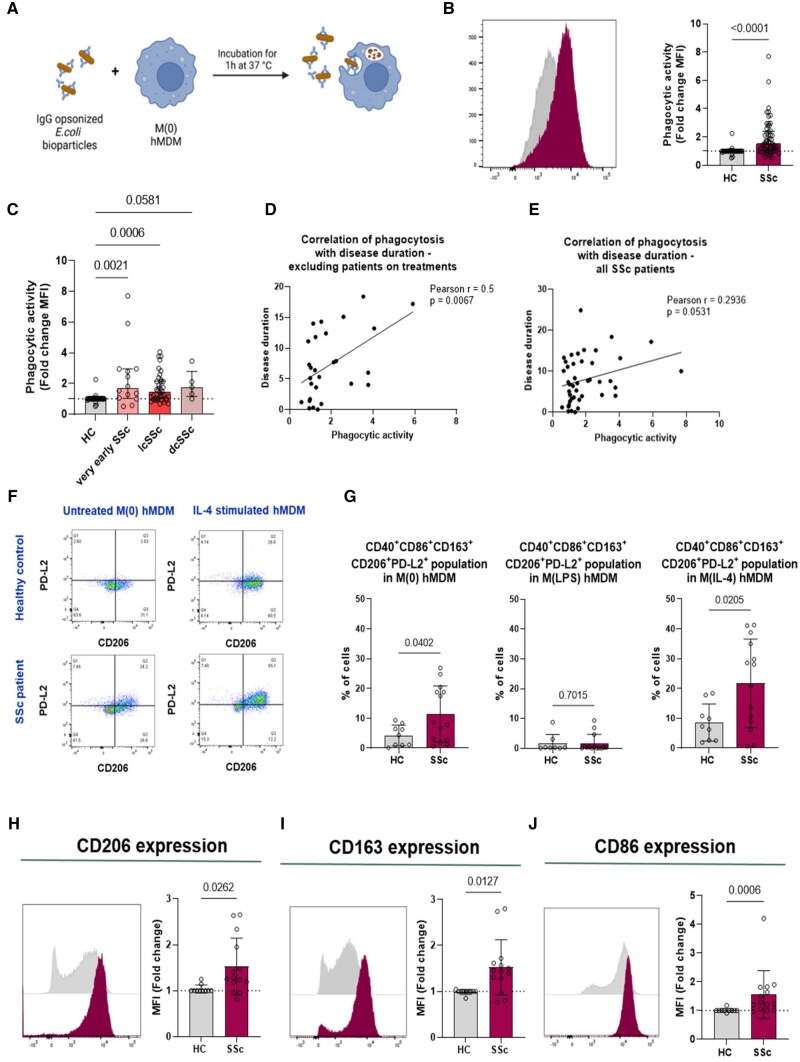
SSc hMDMs display upregulated phagocytosis in all subtypes alongside an alternatively-activated phenotype. (A) Experimental workflow of *in vitro* phagocytosis assessment in M0 hMDMs. CD14^+^ monocytes were isolated from PBMCs from HC and SSc patients and differentiated into hMDMs with rh M-CSF (50 ng/ml) and left unpolarized as M0 hMDMs. (B) M0 HC (*n* = 36) and SSc (*n* = 57) hMDMs were incubated for 1 h with pHrodo bioparticles and the uptake was assessed by flow cytometry. A representative histogram of phagocytic activity (measured as MFI) and corresponding analysis of M0 HC (grey histogram) and SSc (purple histogram) hMDMs. (C) MFI fold change results of phagocytic activity of hMDMs from SSc patients were further divided into the different subtypes and compared. (D) and (E) Macrophage phagocytosis was correlated with disease duration from with patients without current treatments (*n* = 28) (D), or all patients (*n* = 44) (E). F–J, Differentiated HC (*n* = 10) and SSc (*n* = 14) hMDMs were either left unpolarized M0, stimulated with LPS (10 ng/ml) to M(LPS) or IL-4 (10 ng/ml) to M(IL-4). hMDMs were stained with fluorescently labelled surface antibodies and analysed by flow cytometry. (F) Representative gating into PD-L2 and CD206 markers already gated into CD40^+^CD86^+^CD163^+^ cells. (G) Percentages of living CD40^+^CD86^+^CD163^+^PD-L1^+^CD206^+^ cells comparing HC and SSc patients in M0, M(LPS), and M(IL-4) hMDMs. (H–J) Quantifications of MFI (fold changes) of CD206 (H), CD163 (I) and CD86 (J) comparing HC and SSc hMDMs. Data are shown as mean (s.d.) or median ± interquartile range. Significance was determined using unpaired non-parametric Mann–Whitney *U* test, unpaired two-tailed parametric *t* test or Kruskal-Wallis test with Dunn’s multiple comparison. Correlations were assessed by Pearson coefficient analysis. Illustration created with BioRender.com

To determine a functionality of pro-phagocytic macrophages in SSc, we incubated M0 human monocyte-derived macrophages (hMDMs) from patients and HC with pHrodo bioparticles *in vitro*, and measured their uptake by flow cytometry (Fig. 2A). We observed increased phagocytic activity in M0 hMDMs across all SSc subtypes (very early, lcSSc and dcSSc) compared with controls (Fig. 2B and C). This increased activity was present even in very early SSc patients who did not meet ACR/EULAR 2013 classification criteria. A moderate positive correlation (*r* = 0.5, *P* = 0.0067) between phagocytic activity and disease duration was found in treatment-naïve patients (Fig. 2D). No significant correlation (*r* = 0.2936, *P* = 0.0531) was observed when including all patients, indicating potential treatment effects on the phagocytic phenotype (Fig. 2E).

### SSc hMDMs exhibit an alternatively-activated macrophage phenotype

We analysed the characteristics of pro-phagocytic hMDMs in SSc patients by examining phenotypic markers for classically activated M(LPS) and alternatively-activated M(IL-4) macrophages (CD38, CD40, CD86, CD163, CD206, PD-L2, PD-L1) (Supplementary Fig. S3A, available at *Rheumatology* online). We identified seven predominant populations in M0 hMDMs (Supplementary Fig. S3B, available at *Rheumatology* online), noting a mix of classically and alternatively activated phenotypes. We observed an increase in CD40^+^CD163^+^CD86^+^CD206^+^PD-L2^+^ cells in M0 and M(IL-4) SSc hMDMs compared with HC (Fig. 2F and G), though this was not evident in M(LPS) polarized cells (Fig. 2G). Notably, M0 SSc hMDMs exhibited higher expression of CD206 (Fig. 2H) and CD163 (Fig. 2I) markers, along with increased levels of CD86 (Fig. 2J) and CD40 (Supplementary Fig. S3C, available at *Rheumatology* online).

### Actin cytoskeleton reorganization is crucial for efficient phagocytosis in SSc hMDMs

**Figure 3.**
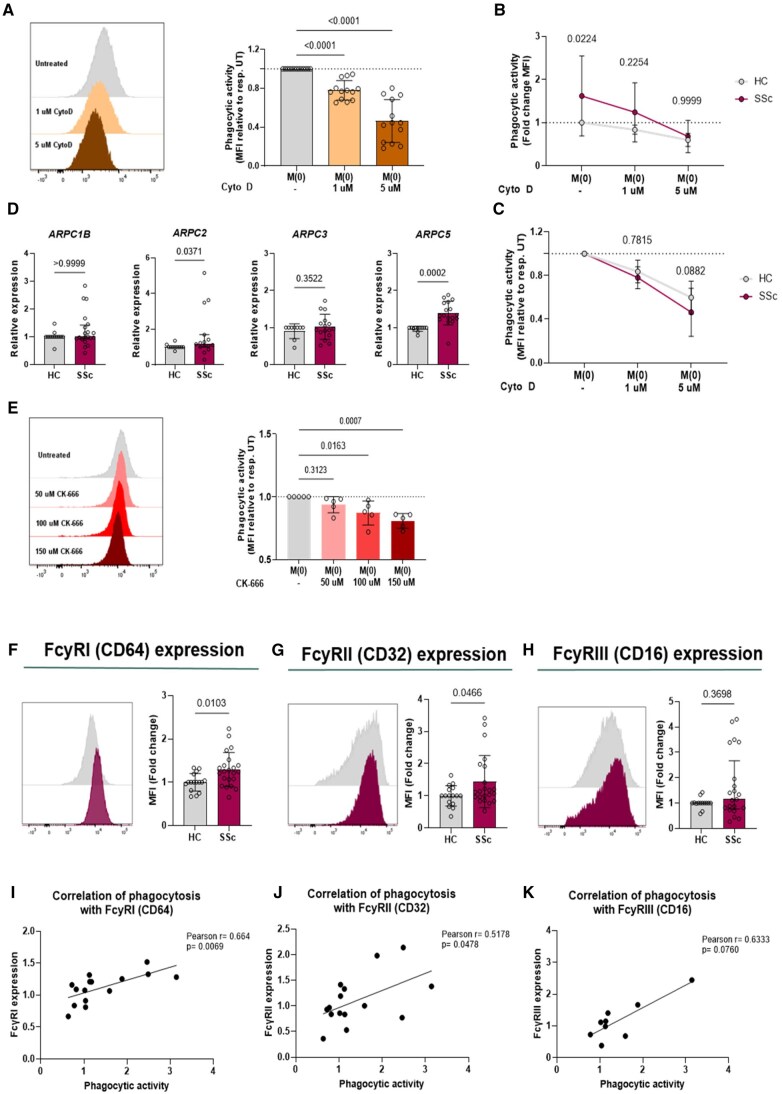
Heightened FcyR expression reveals initial steps in the phagocytic process as drivers of enhanced macrophage phagocytosis observed in SSc. (A) Inhibition efficiency of cytochalasin D in M(0) SSc hMDMs [representative histogram and calculated fold changes (MFI) to untreated are shown, *n* = 13]. Data are presented as mean (s.d.). Significance was determined using one-way ANOVA test with Dunnett’s multiple comparison. (B) Phagocytic activity of untreated and after inhibition with 1 and 5 μM cytochalasin D of M(0) HC (*n* = 10) and SSc (*n* = 13) hMDMs are shown (Fold changes MFI to HC untreated). Data are presented as mean (s.d.). Two-way ANOVA test with Bonferroni’s multiple comparison was performed. (C) Inhibition efficiency of cytochalasin D in M(0) HC and SSc hMDMs is shown (fold changes MFI to HC or SSc untreated, respectively). Data are presented as mean (s.d.). Two-way ANOVA test with Bonferroni’s multiple comparison was performed. (D) Gene expression of *ARPC1B*, *ARPC2*, *ARPC3* and *ARPC5* from HC (*n* = 9–12) and SSc (*n* = 15–20) M(0) hMDMs were measured by RT-qPCR. Data are shown as mean (s.d.) or median ± interquartile range. Significance was determined using unpaired non-parametric Mann-Whitney U test or unpaired two-tailed parametric *t*-test. (E) Inhibition efficiency of CK-666 in pooled M(0) HC and SSc hMDMs [representative histograms and calculated fold changes (MFI) to untreated are shown, *n* = 5]. Data are presented as mean (s.d.) Significance was determined using one-way ANOVA test with Dunnett’s multiple comparison. (F–K) Expression of surface FcγRI (CD64) (F), FcγRII (CD32) (G) and FcγRIII (CD16) (H) of M(0) hMDM from HC (*n* = 14–16) and SSc patients (*n* = 15–22) were assessed by flow cytometry. (I–K) M(0) hMDM phagocytosis results from HC and SSc patients were correlated with the expression of FcγRI (*n* = 14) (I), FcγRII (*n* = 14) (J) and FcγRIII (*n* = 9) (K). Data are shown as mean (s.d.) or median ± interquartile range. Significance was determined using unpaired non-parametric Mann-Whitney *U* test or unpaired two-tailed parametric *t*-test. Correlations were assessed by Pearson coefficient analysis

We conducted *in vitro* inhibition studies to explore the role of the actin cytoskeleton in enhanced macrophage phagocytosis. Using cytochalasin D, a potent actin polymerization inhibitor [20], we observed a dose-dependent reduction in phagocytic activity, with 1 and 5 μM cytochalasin D causing 22% ± 10% and 54% ± 22% inhibition, respectively (Fig. 3A). This indicates the actin cytoskeleton’s critical role in phagocytosis in SSc hMDMs. Pre-treatment with cytochalasin D showed no significant difference in phagocytic activity between M0 SSc and HC hMDMs (Fig. 3B), but 5 μM cytochalasin D led to greater inhibition in SSc hMDMs (54% ± 2.2%) compared with controls (40% ± 1.5%) (Fig. 3C). Transcriptomic analysis revealed enrichment of actin cytoskeleton regulation genes, including *ARPC* family genes (Fig. 1F and I, Supplementary Fig. S2, available at *Rheumatology* online). Consequently, we measured the expression of *ARPC* genes, which encode for the ARP2/3 complex, leading to the nucleation of branched actin filaments [21]. We found increased expression of *ARPC2* and *ARPC5* in M0 SSc hMDMs (Fig. 3D). Additionally, CK-666, an ARP2/3 complex inhibitor [22], caused dose-dependent reductions in phagocytosis with 100 and 150 μM CK-666, leading to 13% ± 9.6% and 19% ± 5.9% inhibition (Fig. 3E), underscoring the ARP2/3 complex's role in phagocytosis. These results confirm that while actin cytoskeleton reorganization is involved in phagocytosis, it may not be the primary driver of increased phagocytic activity in SSc macrophages.

### Upregulated FcγRI and FcyRII on SSc hMDMs correlate with phagocytosis

We further investigated mechanisms driving enhanced macrophage phagocytosis in SSc patients by examining opsonic phagocytic receptors, including FcγRs and complement receptors. We observed significantly increased expression of FcγRI (Fig. 3F) and FcγRII (Fig. 3G), but not FcγRIII (Fig. 3H), in M0 hMDMs from SSc patients compared with HC. Integrin CD18 was also significantly upregulated in M0 SSc hMDMs, whereas CD11b (integrin αM, CR3A) and CD11c (integrin αX, CR4 subunit) were not (Supplementary Fig. S3D, available at *Rheumatology* online). A positive correlation was found between phagocytic activity and FcγRI (Fig. 3I) and FcγRII (Fig. 3J), but not FcγRIII (Fig. 3K), with FcγRI showing the strongest correlation (Pearson *r* = 0.664, *P* = 0.0069) (Fig. 3I). These results suggest that upregulated FcγRs contribute to enhanced phagocytosis in SSc patients.

In pro-inflammatory M(LPS) hMDMs, we observed increased phagocytic activity (Supplementary Fig. S4A and B, available at *Rheumatology* online) along with elevated FcγRI (Supplementary Fig. S4C, available at *Rheumatology* online) and FcγRII (Supplementary Fig. S4D, available at *Rheumatology* online), but not FcγRIII (Supplementary Fig. S4E, available at *Rheumatology* online), compared with HC. CD18 was significantly upregulated in M(LPS) SSc hMDMs, while CD11b (integrin αM, CR3A) and CD11c (integrin αX, CR4 subunit) were not (Supplementary Fig. S4F, available at *Rheumatology* online). M(LPS) SSc hMDMs also exhibited higher levels of pro-inflammatory markers CD86, CD38 and PD-L1 (Supplementary Fig. S5A–D, available at *Rheumatology* online) and increased secretion of TNF-α and IL-10 (Supplementary Fig. S5E, available at *Rheumatology* online). Most cytokines remained undetectable under M0 conditions (Supplementary Fig. S5F, available at *Rheumatology* online). These findings indicate that enhanced phagocytosis in SSc hMDMs, also under classically-activated conditions, is driven by increased FcγR expression.

### Pro-phagocytic SSc tissue macrophages and hMDMs reveal a pro-inflammatory signature with upregulated IL6 expression

**Figure 4.**
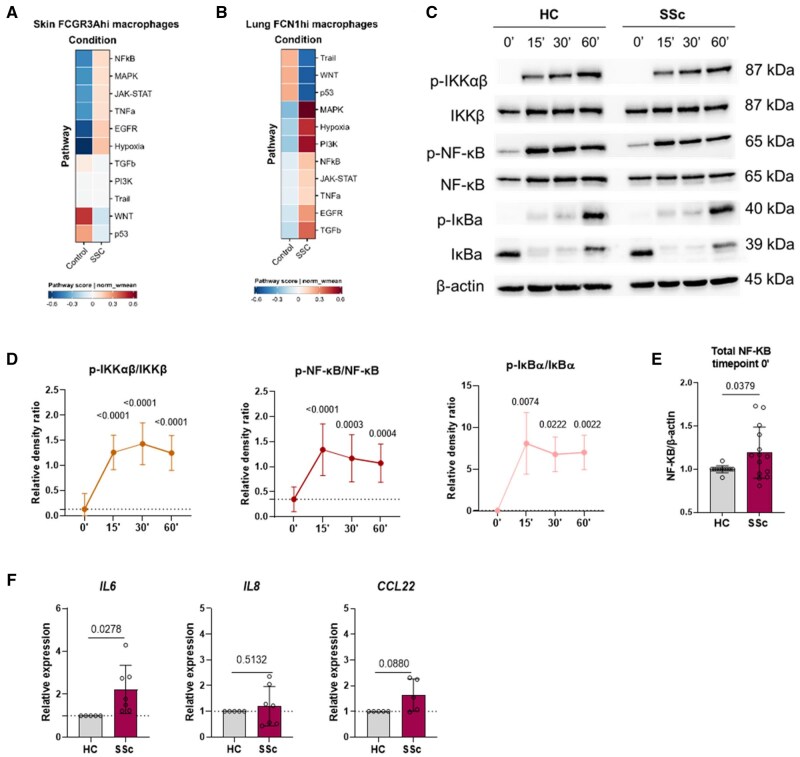
Subsequent pro-inflammatory cascades are induced following phagocytosis. (A) Heatmap of pathway scores is shown comparing *FCGR3A*^hi^ skin macrophages from HC and dcSSc patients. (B) Heatmap of pathway scores is shown comparing *FCN1*^hi^ lung macrophages from HC and SSc-ILD patients. (C) Representative Western Blot of NF-κB pathway assessment at timepoints 0’, 15’, 30’ and 60’ (in min). (D) Quantification of p-IKKαβ/IKKβ, p-NF-κB/NF-κB and p-IκBa/IκBa ratios are shown for SSc hMDMs (*n* = 8–9). (E) Quantification of total NF-κB expression at timepoint 0’ comparing HC (*n* = 12) vs SSc (*n* = 14) M(0) hMDMs. Data are shown as mean (s.d.). Significance was assessed with an unpaired two-tailed parametric *t*-test. (F) 5 h after incubation with pHrodo bioparticles gene expression was assessed by RT-qPCR. *IL6*, *IL8* and *CCL22* expression were measured in M(0) hMDMs from HC (*n* = 5) and SSc (*n* = 6–7) patients. Data are shown as mean (s.d.). One sample *t*-test was used

Transcriptomic analysis showed a strong pro-inflammatory profile in skin *FCGR3A*^hi^ and lung *FCN1*^hi^ macrophages from SSc patients compared with HC (Fig. 4A and B). This pro-inflammatory signature was marked by upregulation of hypoxia, MAPK and NF-κB pathways (Fig. 4A and B). We then investigated whether phagocytosis of pHrodo bioparticles activated the NF-κB signalling cascade in M0 SSc hMDMs. Time-course experiments (0’, 15’, 30’, 60’) revealed strong activation of NF-κB signalling (Fig. 4C and D, Supplementary Fig. S6, available at *Rheumatology* online) and increased total NF-κB expression at baseline (0’) in M0 SSc hMDMs compared with controls (Fig. 4E). Further, phagocytosis led to significantly higher *IL6* expression in SSc hMDMs five hours post-incubation (Fig. 4F). These results confirm that SSc hMDMs exhibit a robust pro-inflammatory response.

### Nintedanib reduces phagocytic activity via impairing FcyRI expression in SSc hMDMs

**Figure 5.**
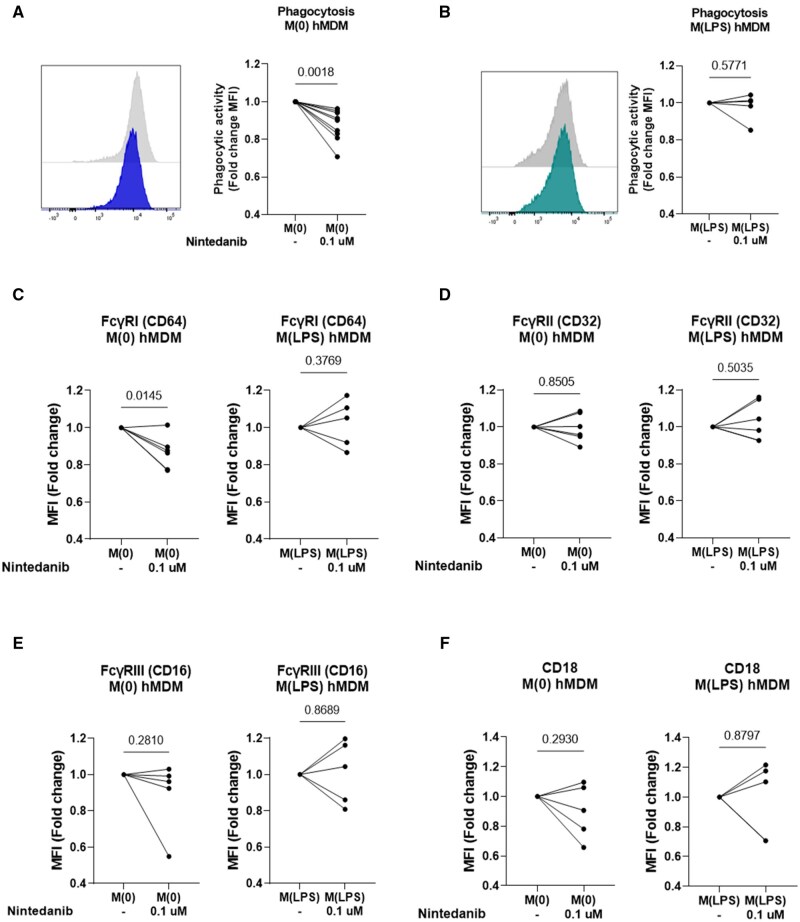
Tyrosine kinase inhibitor nintedanib diminished phagocytic activity of M0 hMDMs, paralleled by a decrease in FcγRI expression. (A–F) SSc hMDMs were treated with 0.1 μM nintedanib or left untreated simultaneously with their polarization into M(0) or M(LPS) for a total of 24 h. (A and B), hMDMs were then incubated for 1 h with pHrodo bioparticles and uptake was assessed by flow cytometry. Representative histogram and quantification of MFI (fold changes) for M(0) (A) or M(LPS) (B). Significance was assessed using a paired *t*-test. Expression of surface FcγRI (CD64) (C), FcγRII (CD32) (D), FcγRIII (CD16) (E) and CD18 (F) of M(0) and M(LPS) hMDM from SSc patients (*n* = 5–6) were assessed by flow cytometry. Significance was assessed using a paired *t*-test

Nintedanib has been reported to reduce M2 macrophage counts and activation in hMDMs and in *Fosl2*^tg^ mice, a model of SSc [23, 24]. However, its role on macrophage phagocytosis has not been investigated. We pre-treated SSc hMDMs with 0.1 μM nintedanib for 24 h, both in M0 and LPS-polarized M(LPS) conditions. Nintedanib significantly reduced phagocytic activity in M0 SSc hMDMs (Fig. 5A), but did not affect phagocytosis in M(LPS) polarized SSc hMDMs (Fig. 5B). Thus, our data indicate that nintedanib can affect phagocytosis of M0 SSc hMDMs but not classically activated M(LPS) SSc hMDMs. Flow cytometry showed no change in surface polarization markers (Supplementary Fig. S7, available at *Rheumatology* online). Additionally, nintedanib reduced FcγRI expression in M0 hMDMs (Fig. 5C), but had no effect on FcγRII (Fig. 5D), FcγRIII (Fig. 5E) or CD18 (Fig. 5F) expression.

### Pro-phagocytic macrophages are also present in other rheumatic autoimmune inflammatory diseases, including RA and PsA

**Figure 6.**
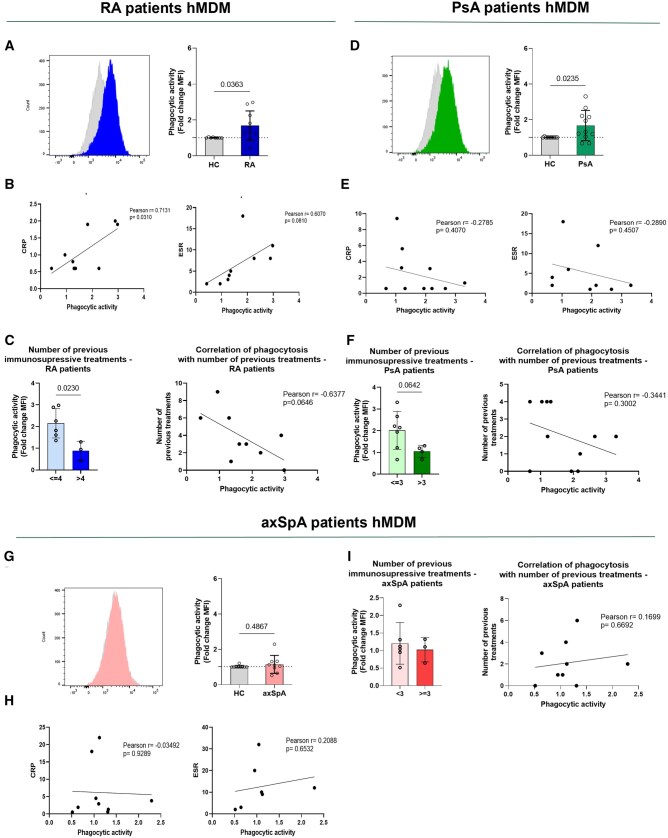
Enhanced macrophage phagocytosis in RA and PsA cohorts. (A–I) CD14^+^ monocytes were isolated from PBMCs from HC (*n* = 8–10), RA patients (*n* = 10), PsA patients (*n* = 11) and axSpA patients (*n* = 9). CD14^+^ monocytes were then differentiated into hMDMs using rh M-CSF (50 ng/ml). M(0) hMDMs were incubated for 1 h with pHrodo bioparticles and uptake was assessed by flow cytometry. A representative histogram of phagocytic activity (measured as MFI) of M(0) hMDMs and corresponding analysis of M(0) HC and RA (A), PsA (D), and axSpA (G) patient hMDMs is shown. Macrophage phagocytosis was correlated with CRP and ESR from RA patients (*n* = 9) (B), PsA patients (*n* = 10) (E), and axSpA patients (*n* = 8) (H). Effects of total number of previous immunosuppressive treatments were assessed from RA patients [≤4, (*n* = 6), >4, (*n* = 3)] (C), PsA patients (≤3, (*n* = 7), >3, (*n* = 4)] (F) and axSpa patients [<3, (*n* = 6), ≥3, (*n* = 3)] (I). Macrophage phagocytosis was correlated with number of previous immunosuppressive treatment from RA patients (*n* = 9) (C), PsA patients (*n* = 11) (F) and axSpA patients (*n* = 9) (I). Data are shown as mean (s.d.). Significance was determined using unpaired two-tailed parametric *t*-test

To determine whether enhanced macrophage phagocytosis is specific to SSc, we analysed phagocytic activity in macrophages from PsA, RA and axSpA patients using pHrodo bioparticles. M0 hMDMs from RA patients exhibited increased phagocytic activity compared with HC (Fig. 6A). There was a strong positive correlation between macrophage phagocytosis and the inflammatory biomarker CRP (Pearson *r* = 0.7131, *P* = 0.0310), and a moderate correlation with ESR (Pearson *r* = 0.6070, *P* = 0.0810) (Fig. 6B). A moderate negative correlation was found between phagocytic activity and the number of previous treatments (Pearson *r* = −0.6377, *P* = 0.0646) (Fig. 6C). Similarly, M0 hMDMs from PsA patients showed increased phagocytosis compared with controls (Fig. 6D), but no significant correlations with CRP, ESR, or treatment history were observed (Fig. 6E and F). Higher treatment numbers (≤3 vs >3) were associated with reduced phagocytic activity (Fig. 6F). In contrast, axSpA patients’ hMDMs did not differ in phagocytic activity from controls (Fig. 6G) and showed no correlations with CRP, ESR or treatments (Fig. 6H and I). M(LPS) hMDMs from RA and PsA patients exhibited increased phagocytosis, while axSpA did not (Supplementary Fig. S8, available at *Rheumatology* online). Using the Idiopathic Pulmonary Fibrosis (IPF) Cell Atlas, we found increased expression of phagocytosis-related genes (*FCGR1A, ARPC4, ARPC5*) in IPF patients across multiple datasets (Supplementary Fig. S9–S11, available at *Rheumatology* online). Additionally, in a second SSc-ILD cohort, elevated expression of *FCGR1A* and *ARPC5* was confirmed (Supplementary Fig. S9, available at *Rheumatology* online).

## Discussion

Dysregulated inflammatory processes can lead to chronic inflammation and fibrosis. This study employed a translational approach to identify FcγR-expressing pro-phagocytic macrophages in SSc, which may drive chronic inflammation. Our *in vitro* findings demonstrate a pro-phagocytic macrophage phenotype not only in SSc but also in other RAIDs, such as RA and PsA, indicating a significant role for enhanced macrophage phagocytosis in inflammatory-driven RAIDs.

This is the first study to investigate macrophage phagocytosis across multiple RAIDs. Previous research has mostly focussed on pulmonary disorders, which typically show impaired phagocytosis [25–27]. Notably, one study reported increased phagocytosis of Herpes Simplex Virus-1 in murine macrophages with an enhanced alternatively-activated M2-like macrophage phenotype [28]. In our study, we identified increased phagocytosis associated with alternatively-activated macrophages and the activation of the pro-inflammatory NF-κB signalling cascade, alongside higher *IL6* expression. This suggests that alternatively activated macrophages might contribute to a chronic pro-inflammatory state in RAIDs.

We observed a significant upregulation of FcγRI and FcγRII in SSc macrophages, correlating positively with increased phagocytic activity. Our data expand on previous research, including a GWAS study, which identified significant loci within the *FCGR* region associated with SSc in the Japanese population [29]. These loci exhibited trends of decreased *FCGR2A* and *FCGR2B* expression in relevant cell types, such as B cells, NK cells, and monocytes. However, our current findings reveal a different aspect of *FCGR* regulation, showing that surface expression of FcyRI and FcyRII is markedly increased on macrophages in SSc patients, with FcγRI being particularly prominent. Recent reports have also identified upregulated expression of FcγRI on monocytes from RA [16], PsA [30] and SLE [15] patients, suggesting their potential role beyond SSc. The upregulation of FcγRI and FcγRII may amplify macrophage-mediated inflammation and tissue damage characteristic of SSc, providing further insight into the pathogenic mechanisms underlying the disease and highlighting the role of Fcγ receptors in autoimmune inflammatory processes.

It is noteworthy that the use of intravenous immunoglobulins (IVIG) has been shown to be beneficial for SSc patients in reducing skin involvement, particularly for those refractory to conventional therapies; however, the exact mechanisms remain uncertain [29]. Our findings of elevated FcγR expression might help explain the therapeutic benefits of IVIG in SSc. Future research should focus on identifying the specific signalling pathways activated downstream of FcγRI and FcγRII in SSc macrophages and explore potential therapeutic interventions. Investigating small molecule inhibitors or monoclonal antibodies targeting FcγRI could provide new therapeutic strategies for SSc. Additionally, several studies, including one from our group, have shown a strong type I IFN signature in SSc monocytes/macrophages [12, 31, 32]. IFN-α has been identified to enhance FcγRI expression on monocytes [15]. Future studies should investigate whether the increased type I IFN signature drives FcγR expression and macrophage phagocytosis in RAIDs.

Phagocytosis in SSc hMDMs was increased in all disease subtypes, not limited to dcSSc patients. This finding contrasts with our transcriptomic analysis of the skin dataset from Gur *et al.* [18]. Our data indicated that the pro-phagocytic signature is a common feature of SSc patients in *in vitro* differentiated hMDMs from peripheral blood, potentially playing a crucial role in multi-organ diseases. Our transcriptomic findings may indicate that tissue-specific factors may play an important role, as only early-stage and dcSSc patients exhibit upregulation of FcγR receptors and associated pathways in skin and lung tissue, while lcSSc patients show no difference of these receptors—potentially influenced by distinct local regulatory factors. The role of the tissue microenvironment appears central to these findings, possibly driving differences in FcγR expression and pro-phagocytic responses across SSc subtypes. Further, it would be of interest to assess the origin of pro-phagocytic macrophages in tissue. Several animal models have shown that recruited monocyte-derived alveolar macrophages are the main contributors to lung fibrosis development [33, 34]. If pro-phagocytic macrophages in dcSSc patients originate from monocyte-derived macrophages, this could be an interesting link to severe organ manifestations in RAID patients.

Cross-sectional data revealed that the number of previous treatments might affect macrophage phagocytosis. Nintedanib, a broad tyrosine kinase inhibitor, is known to reduce M2 macrophage markers (CD163 and CD206) by targeting the Csf1R receptor [23, 24]. Our study found that nintedanib impaired phagocytic activity in alternatively-activated M0 hMDMs, but did not affect the phagocytosis of classically activated M(LPS) hMDMs. While nintedanib did not significantly alter macrophage polarization markers, it did reduce FcγRI expression. Previous research indicated that nintedanib affects M2 macrophage polarization primarily after additional stimulation with anti-inflammatory cytokines like IL-4/IL-13 [23, 24]. Our findings suggest that nintedanib’s impact on phagocytosis might be related to its effect on FcγRI rather than a direct influence on alternatively-activated M0 hMDMs.

Increased expression of *ARPC* subunit genes in M0 SSc hMDMs and our inhibition studies revealed functional actin polymerization as crucial for proper and efficient phagocytosis. Prior research has linked increased ARPC5 expression to enhanced cell migration in cancers, implicating it in immune cell infiltration into tumours [35, 36]. Similarly, our transcriptomic analysis of SSc and IPF patients revealed upregulated *ARPC* gene expression, suggesting that an active actin cytoskeleton network may facilitate the migration of pro-phagocytic monocyte-derived macrophages into organs affected by multi-organ diseases.

The observed differences among RAIDs can be understood through the continuum hypothesis of immunology, which positions diseases along a spectrum ranging from autoimmunity to autoinflammation [37]. Future research should explore whether variations in phagocytic responses across different RAIDs are driven by more pronounced autoimmune mechanisms. To test this hypothesis, additional studies should include other rheumatic diseases that distinctly represent each end of the spectrum, such as SLE (a classical autoimmune disease) and gout (a classical autoinflammatory disorder) [38].

In summary, this study provides a new perspective on macrophage phagocytosis as a potential driver of inflammatory responses in SSc and other RAIDs. The identification of a pro-phagocytic macrophage phenotype indicates a common pathway in various immune-mediated inflammatory conditions. Enhanced surface expression of FcγRI and FcγRII, coupled with increased phagocytosis, underscores the central role of Fcγ receptors in SSc pathogenesis. Notably, our findings particularly highlight FcγRI as a critical player, suggesting it as a potential therapeutic target to alleviate inflammation and tissue damage in affected patients. These findings pave the way for future research and possible clinical interventions in SSc.

## Supplementary Material

keae688_Supplementary_Data

## Data Availability

The data underlying this article will be shared upon request to the corresponding author.
