# Supplementary material for: Elevated Fcy receptor expression augments pro-inflammatory macrophage phagocytosis in systemic sclerosis and associated rheumatic diseases
Source: Rheumatology (Oxford). 2024 Dec 13;64(6):3975–88. doi: 10.1093/rheumatology/keae688 (PMC12107054; doi:10.1093/rheumatology/keae688)
Supplement: keae688_Supplementary_Data [file keae688_supplementary_data.zip › keae688_Supplementary_Data/rhe-24-2041-File002.docx]

## Online Supplementary Material

## Methods – Supplementary Data S1

### Isolation of human CD14^+^ monocytes and differentiation to hMDM

Blood samples were collected in EDTA tubes (BD Vacutainer). Whole blood was diluted two times in PBS and layered on top of cell separation media (Lympholyte, Cedarlane). Diluted blood samples were centrifuged at 800g for 20 minutes with breaks-off. The buffy coat was carefully collected and washed once with FACS buffer composed of PBS containing 1% FBS (Gibco) and 500 μM EDTA (Gibco). Isolated PBMC were then incubated with CD14 Microbeads (Miltenyi Biotec) for 15 minutes at 4°C. Subsequently, stained cells were washed with FACS buffer, resuspended in 2 ml FACS buffer and applied on the autoMACS Pro Separator (Miltenyi Biotec). CD14^+^ monocytes were separated using the predefined “Possel” program. Positive selected cells were counted and washed once with complete RPMI medium (Gibco) supplemented with 10% FBS and 1% Penicillin/Streptomycin (Gibco). Finally, CD14^+^ monocytes were resuspended in complete RPMI medium containing 50 ng/ml recombinant human (rh) M-CSF (Immunotools), plated at the desired concentration in cell culture plates, and kept at 37°C and 5% CO_2_. Monocytes were differentiated for a total of 8 days into hMDM with a medium change after 4 days. For *in vitro* experiments hMDM were either left unpolarized M(0) or further polarized with 10 ng/ml LPS (Sigma-Aldrich) to M(LPS) or 10 ng/ml rh IL-4 (Immunotools) to M(IL-4).

### *In vitro* phagocytosis assay

Phagocytic activity of hMDM was assessed using pHrodo Red Bioparticles Conjugate for Phagocytosis (Invitrogen). First, fluorescently labeled bacteria were reconstituted with PBS and then incubated with equal volume of BioParticles Opsonizing Reagent (Invitrogen) for 1 hour at 37°C. Bioparticles were then washed three times with PBS using low-speed centrifugation at 1500g for 15 minutes. Opsonized pHrodo Red bioparticles were then resuspended in PBS to a final concentration of 1 mg/ml. Unpolarized M(0) or LPS polarized M(LPS) hMDM (1x10^6^ cells) were incubated with 50 ug pHrodo Red bioparticles for 1 hour at 37°C. For some experiments, hMDM were treated with 0.1 μM nintedanib (Selleckchem) 24 hours before the addition of pHrodo Red bioparticles. For phagocytosis inhibition studies, hMDM were pre-treated for 30 min before the addition of pHrodo Red bioparticles with CK-666 (50-150 μM; Sigma-Aldrich) or Cytochalasin D (1-10 μM; Sigma-Aldrich). For harvesting, cells were washed with PBS and detached using Accutase (Biolegend). Complete RPMI medium was used to block enzyme activity and cells were washed twice with ice-cold PBS. In a last step, hMDM were stained with eBioscience Fixable Viability Dye eFluor 780 (Invitrogen) or Zombie Yellow (Biolegend) dye for live/dead discrimination. Samples were acquired using the BD LSRFortessa II flow cytometer and median fluorescence intensities (MFI) were determined in FlowJo 10.6.2 software.

### Flow cytometry

In vitro cultured hMDM were first washed with PBS and then detached using Accutase. Complete RPMI medium was used to block enzyme activity and cells were washed with FACS buffer. To block unspecific binding, hMDM were incubated with True-Stain Monocyte Blocker (Biolegend) for 10 minutes at 4 °C, followed by the subsequent incubation of specific fluorochrome-conjugated anti-human antibody mixes for 20 minutes. The following anti-human antibodies were used specific to: CD16 (FcγRIII) (clone 3G8, APC, Biolegend, 1:200); CD32 (FcγRII) ((clone FUN-2, PE, Biolegend, 1:100), (clone 6C4 (CD32), FITC, Biolegend, 1:100)); CD64 (FcγRI) ((clone 10.1, APC, Biolegend, 1:100), (clone 10.1, PE, Biolegend, 1:100)); CD11b (clone ICRF44, APC, Biolegend, 1:200); CD11c (clone 3.9, PE, Biolegend, 1:100); CD18 (clone TS1/18, FITC, Biolegend, 1:100); CD38 (clone HB-7, PE/Dazzle, Biolegend, 1:400); CD40 (clone 5C3, AlexaFluor700, Biolegend, 1:400); CD86 (IT2.2, APC Biolegend, 1:400); PD-L1 (clone MIH3, FITC, Biolegend; 1:200), CD206 (clone 15-2, PE-Cy5, Biolegend, 1:800); and CD163 (clone GHI/61, PE, Biolegend, 1:200).

After antibody staining, cells were washed twice with PBS. Next, for live/dead cell discrimination, hMDM were stained with eBioscience Fixable Viability Dye eFluor 780 for 20 minutes at 4 °C. Finally, samples were acquired at BD LSRFortessa II flow cytometer and analyzed using the FlowJo 10.6.2 software. For compensation, single stained samples were used and FMO controls were included in the experiments.

### Real-time quantitative PCR (RT–qPCR) with reverse transcription

Total RNA was isolated using the Quick-RNA MicroPrep Kit (Zymo Research) following the manufacturer’s instruction. RNA concentration and purity was measured using the NanoDrop 2000 (Thermo Fisher Scientific). Total RNA was reverse transcribed into cDNA using using MultiScribe reverse transcriptase (Thermo Fisher Scientific). Afterwards, qPCR was performed using the SYBR green GoTaq qPCR master mix (Promega) on an Agilent Technologies Stratagene Mx3005P qPCR instrument. Each sample was measured in duplicates. Relative gene expression was calculated using the 2-∆∆Ct method. As a reference gene the geometric mean of CT values of hypoxanthine phosphoribosyltransferase 1 (*HPRT1*) and ribosomal protein lateral stalk subunit P0 (*RPLPO*) was used. Primer sequences are provided below.

Human primer sequences (5’-3’):

*HPRT1* 🡪 Fw: CCT GGC GTC GTG ATT AGT GA, Rv: CGA GCA AGA CGT TCA GTC CT

*RPLP0* 🡪 Fw: ACACTGGTCTCGGACCTGAGAA, Rv: AGCTGCACATCACTCAGAATTTCA

*CCL22* 🡪 Fw: GCG TGG TGT TGC TAA CCT TC, Rv: CCA CGG TCA TCA GAG TAG GC

*IL6* 🡪 Fw: GGG TCA GGG GTG GTT ATT GC, Rv: CAG ATT TGA GAG TAG TGA GGA ACA

*IL8* 🡪 Fw: TAC TCC AAA CCT TTC CAC CCC, Rv: CCC AGT TTT CCT TGG GGT CC

*ARPC1B* 🡪 Fw: CAA GGA CCG CAC CCA GAT T, Rv: TGC CGC AGG TCA CAA TAC G

*ARPC2* 🡪 Fw: GCA GAT TTC GAT GGG GTC CTC, Rv: ACT CCC GTA CAC CCT CTT TAA T

*ARPC3* 🡪 Fw: GTG CAA TTC CAA AAG CCA AGG, Rv: GGC TCT CAT CAC TTC ATC TTC C

*ARPC5* 🡪 Fw: TGG TGT GGA TCT CCT AAT GAA GT, Rv: CAC GAA CAA TGG ACC CTA CTC

### ELISA

Secreted cytokines were measured in cell culture supernatants using Human IL-6, IL-10, and TNF-α ELISA DuoSet kits as described by the manufacturer’s instructions (all from R&D). Briefly, 96 well plates were coated with capture antibodies overnight at RT and in a next step blocked with 1% BSA in PBS. Between each subsequent step, the plates were washed three times with Wash Buffer (0.05% Tween 20 in PBS). Recombinant human cytokine standards and the samples were incubated then for 2 hours at RT. Next, plates were incubated with biotinylated detection antibodies for another 2 hours at RT, followed by the incubation with streptavidin-HRP for 20 min at RT. For signal development, a TMB substrate (BD Biosciences) was used, and the reaction was stop with 2N H_2_SO_4_ solution. Absorbance was measured using a BioTEK HT plate reader set at 450 nm with wavelength correction at 570 nm. Each sample was measured in duplicates and the concentrations were determined according to the respective standard curves.

### Protein extraction and Western Blotting

Unpolarized M(0) hMDM were harvested and washed once with ice-cold PBS. Cells were lysed in RIPA buffer (Sigma) supplemented with protease inhibitors (cOmplete ULTRA tablets, Roche) and phosphatase inhibitors (PhosStop, Roche). Total protein concentration was measured with BCA protein assay according to manufacturer’s instruction (Thermo Scientific). A total of 25 μg protein was loaded and separated by SDS-PAGE electrophoresis. Directly after the separation, proteins were transfer by wet transfer on a nitrocellulose membrane (GE Healthcare). Next, the membranes were incubated with blocking solution consisting of 5% skim milk (BD Life Sciences) in TBST buffer. Membranes were then incubated overnight with primary antibodies in blocking solution or 5% BSA in TBST at 4°C. The following primary anitbodies were used: Anti-IKKβ (clone D30C6, Cell Signaling Technologies, 1:1000); Anti-p-IKKα/β (clone 16A6, Cell Signaling Technologies, 1:1000); Anti-NF-κB p65 (clone D14E12, Cell Signaling Technologies, 1:1000); Anti-p- NF-κB (clone 93H1, Cell Signaling Technologies, 1:1000); Anti-IκBα (clone L35A5, Cell Signaling Technologies, 1:1000); Anti-p-IκBα (clone 14D4, Cell Signaling Technologies, 1:1000); and Anti- β-actin (clone 8H10D10, Cell Signaling Technologies, 1:5000). Afterwards, membranes were washed 3x with TBST and incubated with a secondary antibody for 1 hour at RT. The following secondary antibodies were used: Goat anti-mouse IgG HRP (clone polyclonal, Jackson ImmunoResearch, 1:10000); Donkey anti-rat IgG HRP (clone polyclonal, Jackson ImmunoResearch, 1:10000); Goat anti-rabbit IgG HRP (clone polyclonal, Jackson ImmunoResearch, 1:10000). Membranes were washed again 3x with TBST. For signal detection, ECL substrate (SuperSignal West Pico PLUS, Thermo Scientifc) was applied on the membrane and signal intensity detected using a Fusion fx (Vilber) instrument. Band density was quantified using ImageJ software and fold changes were calculated after normalization to β-actin densitometry.

### Transcriptomic analysis of skin and lung scRNAseq datasets

scRNAseq data for lung and skin samples from SSc patients, as well as healthy controls, were obtained from datasets (GSE138669 for the skin dataset and GSE212109 for the lung dataset).

Count matrices were imported into R as Seurat objects using the Seurat v4.3.0 package [47]. scDblFinder (v1.12.0) was used to exclude doublets from the individual sample matrices [48]. The expression matrices were merged into a single Seurat object and subjected to quality control processing. Cells in the lung dataset were filtered for mitochondrial reads <12%, nFeatures_RNA >800 and nCount_RNA >2000; cells in the skin dataset were filtered for mitochondrial reads <12%, nFeatures_RNA >400 and nCount_RNA >1200.

Datasets were split into individual sample matrices and normalized using Seurat’s SCTransform function [49]. Normalized expression matrices were merged, and dimensionality reduction was performed on the top 3000 most variable genes using principal component analysis (PCA). Datasets were then integrated with Harmony (v 0.1.1) using as covariates the sample, and only for the skin dataset, the 10X Genomics chemistry (3’ V1 or V2) [50].

Clustering was performed using Seurat’s RunUMAP, FindNeighbours and FindCluster functions. The first 25 principal components and a resolution of 0.3 were used for the lung dataset, while 28 principal components and a resolution of 0.4 were used for the skin dataset. Markers for each cluster were identified with the FindAllMarkers function using the default Wilcoxon test, a logFC threshold of 0.5 and 50% as the minimum percentage of cells expressing each gene. Cell-type identity was assigned to each cluster according to these markers.

Clusters annotated as macrophages or dendritic cells were subsetted and reclustered to identify subpopulations. 10 principal components and a resolution of 0.2 were used for macrophages/dendritic cells in the lung dataset; 30 principal components and a resolution of 0.7 were used for macrophages/dendritic cells in the skin dataset. The FindAllMarkers function was again used to identify cluster markers.

Differential expression analysis was performed comparing SSc to healthy control samples in each macrophage subpopulation using the FindMarkers function with default parameters. For GO and pathway analysis the ShinyGO application was used [51].

We inferred pathway activity using the function computePathwayActivityAnalysis from the package ezRun (v3.18.1) developed at the Functional Genomics Center Zurich (FGCZ). This function uses PROGENy as the resource for pathway signatures, target genes and weights for each interaction [52]. Activity scores are then computed as normalized weighted means for each pathway source across cells using the package decoupleR (v2.8.0).

For the second skin dataset analysis, we downloaded the massively-parallel single-cell RNA sequencing (MARS-seq) data available in the GEO database (GSE195452). We stored the expression data together with Meta information (cell types, patient infos, etc.) as a Seurat object. We analysed the cells originating from skin biopsies using the protein coding genes. The data was processed following the standard Seurat workflow with log-normalization, variable genes identification, data reduction using PCA and Uniform Manifold Approximation and Projection (UMAP) [47]. Cell type information was used as provided by the original analysis by Gur et al [22]. We generated pseudobulk data by summarising for each subject the expression profiles of the different cell types. For the macrophage pseudobulk profiles, we computed differential expression between SSc and healthy control individuals. We included only subjects that had at least 3 cells classified as a macrophage. We used the logistic regression test in the Seurat package to compute the differentially expressed genes (DEG).

## Patient demographic and clinical characteristics

| Supplementary Table S1 SSc Patients’ characteristics at baseline | | | | | |
| --- | --- | --- | --- | --- | --- |
|  | All  (N=57) | Very Early SSc  (N=14) | Limited SSc  (N=38) | Diffuse SSc  (N=5) |  |
| Age (mean±SD years) | 52.5±14.0 | 52.1±12.9 | 52.5±14.3 | 53.6±17.0 |  |
| Gender |  |  |  |  |  |
| Female (n/N, %) | 52/57 (91.2) | 13/14 (92.9) | 34/38 (89.5) | 5/5 (100.0) |  |
| Disease duration (median, IQR, years) | 7.5 (3.8-12.6) | 2.7 (0.9-8.6) | 7.9 (4.8-13.7) | 7.6 (4.5-8.0) |  |
| ACR/EULAR 2013 criteria fulfilled (n/N, %) | 43/57 (75.4) | 0/14 | 38/38 (100.0) | 5/5 (100.0) |  |
| mRSS at baseline visit (x/51) (median, IQR) | 0 (0-2) | 0 (0-0) | 0 (0-2) | 4 (3-12) |  |
| Raynaud’s phenomenon ever (n/N, %) | 56/57 (98.2) | 13/14 (92.9) | 38/38 (100.0) | 5/5 (100.0) |  |
|  |  |  |  |  |  |
| Puffy fingers (ever) (n/N, %) | 40/53 (75.4) | 7/12 (58.3) | 30/37 (81.1) | 3/4 (75.0) |  |
|  |  |  |  |  |  |
| Digital ulcers (ever) (n/N, %) | 16/57 (28.1) | 0/14 | 13/38 (34.2) | 3/5 (60.0) |  |
| Digital ulcers (current) (n/N, %) | 5/57 (8.8) | 0/14 | 4/38 (10.5) | 1/5 (20.0) |  |
| Pitting scars on fingertips ever (n/N, %) | 19/56 (33.9) | 0/13 | 16/38 (42.1) | 3/5 (60.0) |  |
| Pitting scars on fingertips (current) (n/N, %) | 13/57 (22.8) | 0/14 | 11/38 (28.9) | 2/5 (40.0) |  |
| Joint synovitis (n/N, %) | 8/55 (14.5) | 2/13 (15.4) | 6/38 (15.8) | 0/4 |  |
| Joint contractures (n/N, %) | 10/54 (18.5) | 0/12 | 8/38 (21.1) | 2/4 (50.0) |  |
| Tendon friction rubs (n/N, %) | 2/57 (3.5) | 0/14 | 2/38 (5.2) | 0/5 |  |
| Muscle weakness (n/N, %) | 1/55 (1.8) | 0/12 | 1/38 (2.6) | 0/5 |  |
| Organ involvement |  |  |  |  |  |
| Lung |  |  |  |  |  |
| Dyspnea NYHA |  |  |  |  |  |
| I (n/N, %) | 32/42 (76.1) | 5/6 (83.3) | 26/33 (78.8) | 1/3 (33.3) |  |
| II (n/N, %) | 9/42 (21.4) | 0/6 | 7/33 (21.2) | 2/3 (66.6) |  |
| III (n/N, %) | 1/42 (2.4) | 1/6 (16.6) | 0/33 | 0/5 |  |
| IV (n/N, %) | 0/42 | 0/6 | 0/33 | 0/5 |  |
|  |  |  |  |  |  |
| Pulmonary arterial hypertension by RHC (mPAP>20mmHg and PWP<15 mmHg) (n/N, %) | 0/0 | 0/0 | 0/0 | 0/0 |  |
| Lung fibrosis on HRCT (n/N, %) | 22/56 (39.2) | 2/13 (15.4) | 16/37 (43.2) | 4/5 (80.0) |  |
| DLCO, lung function test (mean±SD % predicted) | 73.1±19.5 | 77.3±13.0 | 72.9±20.3 | 64.2±27.4 |  |
| DLCO<70% predicted | 25/56 (44.6) | 3/13 (23.1) | 19/38 (50.0) | 3/5 (60.0) |  |
| FVC, lung function test (mean±SD % predicted) | 94.1±15.3 | 98.9±14.2 | 93.6±14.6 | 86.2±22.0 |  |
| FVC<70% predicted | 3/56 (5.3) | 0/13 | 2/38 (5.3) | 1/5 (20.0) |  |
| TLC, lung function test (mean±SD % predicted) | 103.2±17.4 | 104.9±12.5 | 103.6±17.6 | 95.6±27.6 |  |
| TLC <70% predicted | 3/55 (5.5) | 0/13 | 1/37 (2.7) | 2/5 (40.0) |  |
|  |  |  |  |  |  |
| Gastrointestinal tract |  |  |  |  |  |
| Esophageal symptoms (n/N, %) | 22/57 (38.6) | 3/14 (21.4) | 15/38 (39.5) | 4/5 (80.0) |  |
| Stomach symptoms (n/N, %) | 10/56 (17.8) | 1/13 (7.7) | 8/38 (21.1) | 1/5 (20.0) |  |
| Intestinal symptoms (n/N, %) | 14/56 (25.0) | 1/13 (7.7) | 12/38 (31.6) | 1/5 (20.0) |  |
| Cardiovascular system |  |  |  |  |  |
| Conduction blocks (n/N, %) | 4/56 (7.1) | 2/14 (15.4) | 2/37 (5.4) | 0/5 |  |
| LVEF% (mean±SD) | 59.3±4.7 | 60.0±3.5 | 59.3±4.7 | 57.4±7.7 |  |
| LVEF <45% (n/N, %) | 1/56 (1.8) | 0/14 | 0/36 | 1/5 (20.0) |  |
| Diastolic dysfunction (n/N, %) | 3/45 (6.6) | 0/12 | 3/30(10.0) | 0/3 |  |
| Kidneys |  |  |  |  |  |
| Renal crisis (n/N, %) | 1/57 (1.8) | 0/14 | 1/38 (2.6) | 0/5 |  |
| Nailfoldcapillaroscopy (NFC) |  |  |  |  |  |
| Scleroderma pattern (n/N, %) | 39/55 (70.9) | 3/14 (21.4) | 32/36 (88.9) | 4/5 (80.0) |  |
| Early (n/N, %) | 11/33 (33.3) | 0/2 | 10/28 (35.7) | 1/3 (33.3) |  |
| Active (n/N, %) | 12/33 (36.4) | 2/2 (100) | 10/28 (35.7) | 0/3 |  |
| Late (n/N, %) | 10/33 (30.3) | 0/2 | 8/28 (28.6) | 2/3 (33.3) |  |
| Laboratory parameters |  |  |  |  |  |
| ANA (n/N, %) | 54/54 (100) | 13/13 (100) | 37/37 (100.0) | 4/4 (100.0) |  |
| Anti-centromere (n/N, %) | 29/52 (55.8) | 7/13 (53.8) | 22/36 (61.1) | 0/4 |  |
| Anti-Scl-70 (n/N, %) | 9/50 (18.0) | 0/12 | 7/35 (20.0) | 2/4 (50.0) |  |
| Anti-RNA-polymerase III (n/N, %) | 2/50 (4.0) | 0/12 | 1/35 (2.9) | 1/4 (25.0) |  |
| CRP elevation (n/N, %) | 2/57 (3.5) | 0/14 | 1/38 (2.8) | 1/5 (20.0) |  |
| Erythrocyte sedimentation rate elevation (n/N, %) | 7/51 (13.7) | 1/10 (10) | 6/36 (16.6) | 0/5 |  |
| CK elevation (n/N, %) | 5/57 (8.8) | 2/14 (14.3) | 3/38 (7.9) | 0/5 |  |
| Proteinuria (n/N, %) | 0/54 | 0/13 | 0/36 | 0/5 |  |
| Activity VAI>3 | 0/56 | 0/14 | 0/37 | 0/5 |  |
| Immunosuppressive treatment (n/N, %) | 17/57 (29.8) | 1/14 (7.1) | 13/38 (34.2) | 3/5 (60.0) |  |
| Abatacept | 2/57 (3.5) | 0/14 | 2/38 (5.3) | 0/5 |  |
| Azathioprine (n/N, %) | 1/57 (1.8) | 0/14 | 1/38 (2.6) | 0/5 |  |
| Chloroquine/Hidroxychloroquine (n/N%) | 6/57 (10.5) | 1/14 (7.1) | 4/38 (10.5) | 1/3 (33.3) |  |
| Corticosteroid (n/N, %)s | 0/57 | 0/14 | 0/38 | 0/5 |  |
| Cylosporine A | 0/57 | 0/14 | 0/38 | 0/5 |  |
| Cyclophosphamide | 0/57 | 0/14 | 0/38 | 0/5 |  |
| D-penilcillamine | 0/57 | 0/14 | 0/38 | 0/5 |  |
| Leflunamide (n/N, %) | 0/57 | 0/14 | 0/38 | 0/5 |  |
| Methotraxate (n/N, %) | 3/57 (5.2) | 0/14 | 3/38 (7.9) | 0/3 |  |
| Mycophenolate mofetil (n/N, %) | 3/57 (5.2) | 0/14 | 2/38 (5.3) | 1/3 (33.3) |  |
| Nintedanib (n/N, %) | 0/57 | 0/14 | 0/38 | 0/5 |  |
| Rituximab (n/N, %) | 3/57 (5.2) | 0/14 | 2/38 (5.3) | 1/3 (33.3) |  |
| TNF alpha inhibitors | 1/57 (1.8) | 0/14 | 1/38 (2.6) | 0/5 |  |
| Tocilizumab (n/N, %) | 3/57 (5.2) | 0/14 | 2/38 (5.3) | 1/3 (33.3) |  |
| All variables are presented either as mean ± standard deviation (SD), by normal distribution, or as median and 25^th^-75^th^ percentile (Q_1_-Q_3_) if non-Gaussian distribution; categorical variables are shown as frequencies/available cases and valid percentages. Clinical parameters were defined according to the EUSTAR (Meier, F.M., et al., Update on the profile of the EUSTAR cohort: an analysis of the EULAR Scleroderma Trials and Research group database. Ann Rheum Dis, 2012. 71(8): p. 1355-60.)  Abbreviations: ANA-anti-nuclear antibodies; ACA-anti-centromere antibodies; anti-Scl-70-anti-topoisomerase antibodies; Anti-RNA-pol III- anti RNA polymerase III antibodies; CRP-C-reactive protein; ESR-erythrocyte sedimentation rate; CK-creatine kinase; ILD-interstitial lung disease; HRCT-high resolution tomography; FVC-forced vital capacity, % predicted; DLCO-diffusing capacity for carbon monoxide; NYHA-New York Heart; LVEF- left ventricular ejection fraction; VAI- Valentini activity index;  Immunosuppressive treatment- prednisone≥10 mg/day, any DMARDs (methotrexate, leflunamide, cyclophosphamide, hydroxychloroquine, cyclosporine A, azathioprine, mycophenolate mofetil, D-penicillamine), abatacept (fusion protein) or small molecules (Nintedanib) within 2 months and biologics (rituximab within 6 months, tocilizumab, TNFα inhibitors within 3 months). | | | | |  |

| Supplementary Table S2 Other RMD Patients’ characteristics at baseline (PsA, RA and axSpA patients) for correlations with phagocytosis | | |
| --- | --- | --- |
|  | PsA | RA |
|  | (N=11) | (N=10) |
| Age |  |  |
| Mean (SD) | 42.5 (12.1) | 49.2 (14.6) |
| Median [Min, Max] | 46.0 [24.0, 56.0] | 47.5 [30.0, 74.0] |
| Sex |  |  |
| female | 5 (45.5%) | 8 (80.0%) |
| male | 6 (54.5%) | 2 (20.0%) |
| Disease_duration |  |  |
| Mean (SD) | 7.00 (8.44) | 13.7 (8.97) |
| Median [Min, Max] | 4.00 [0, 23.0] | 11.5 [3.00, 34.0] |
| Seropositivity |  |  |
| NA | 3 (27.3%) | 0 (0%) |
| no | 8 (72.7%) | 3 (30.0%) |
| yes | 0 (0%) | 7 (70.0%) |
| HLAB27 |  |  |
| NA | 7 (63.6%) | 9 (90.0%) |
| no | 3 (27.3%) | 1 (10.0%) |
| yes | 1 (9.1%) | 0 (0%) |
| Active_disease |  |  |
| no | 8 (72.7%) | 9 (90.0%) |
| yes | 3 (27.3%) | 1 (10.0%) |
| CRP |  |  |
| Mean (SD) | 2.38 (2.85) | 1.95 (2.72) |
| Median [Min, Max] | 0.600 [0.600, 9.40] | 0.900 [0.600, 9.50] |
| Current_Treatment |  |  |
| MTX | 1 (9.1%) | 4 (40.0%) |
| IL17i | 1 (9.1%) | 0 (0%) |
| IL23i | 3 (27.3%) | 0 (0%) |
| JAKi | 1 (9.1%) | 4 (40.0%) |
| TNFi | 5 (45.5%) | 2 (20.0%) |
| No | 0 (0%) | 0 (0%) |
| No_prevtreatments |  |  |
| Mean (SD) | 2.00 (1.79) | 3.40 (2.91) |
| Median [Min, Max] | 2.00 [0, 4.00] | 3.00 [0, 9.00] |

| All variables are presented either as mean ± standard deviation (SD) or as median and Min, Max. | | |  |  |
| --- | --- | --- | --- | --- |
| Categorical variables are shown as frequencies/available cases and valid percentages. | |  |  |  |
|  |  |  |  |  |
| Abbreviations: NA: not annotated; HLAB27: Human leukocyte antigen B27; CRP-C-reactive protein; | | |  |  |
| Current immunosuppressive treatments: Methotrexate (MTX), IL-17 inhibitors (secukinumab), IL-23 inhibitors (guselkumab), | | | |  |
| JAK inhibitors (baricitinib, upadacitinib), TNFα inhibitors (adalimumab, certolizumab, golimumab), None | | |  |  |
|  |  |  |  |  |
| Active diseases definition: For RA and PsA active disease was defined as DAS28-CRP > 3.2 and for axSpA ASDAS ≥ 2.1 | | | |  |
| Seropositivity: indicates either rheumatoid factor (RF) or anti-citrullinated protein antibodies (ACPA), or both. | | |  |  |

## Supplementary Figures


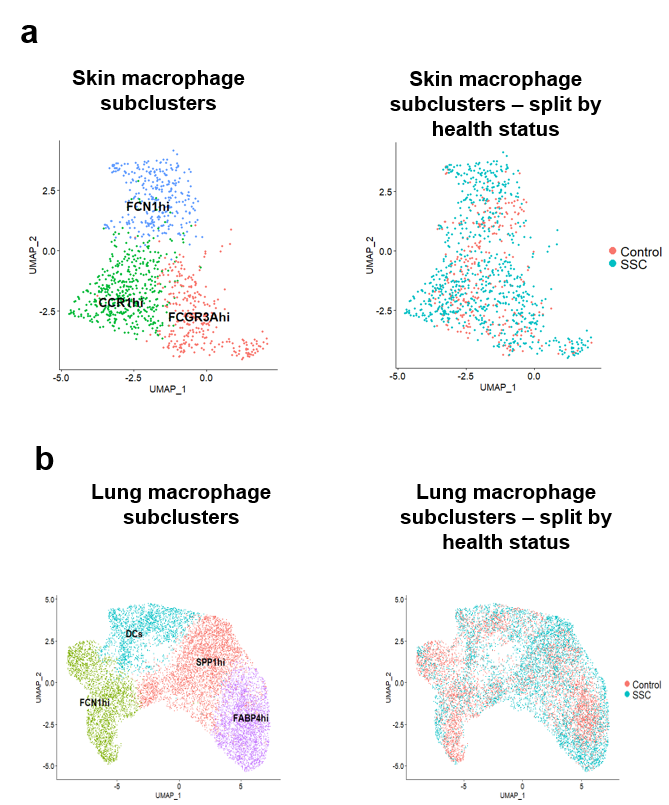


**Supplementary Fig. S1: UMAP plots of skin and lung macrophage subclusters.**

**a,** UMAP plot of the three main skin macrophage subclusters is shown for all samples and when additionally split by health status (healthy control vs dcSSc cells). **b,** UMAP plot of the three lung macrophage subclusters is shown for all samples and when additionally split by health status (healthy control vs SSc-ILD cells).

**a**


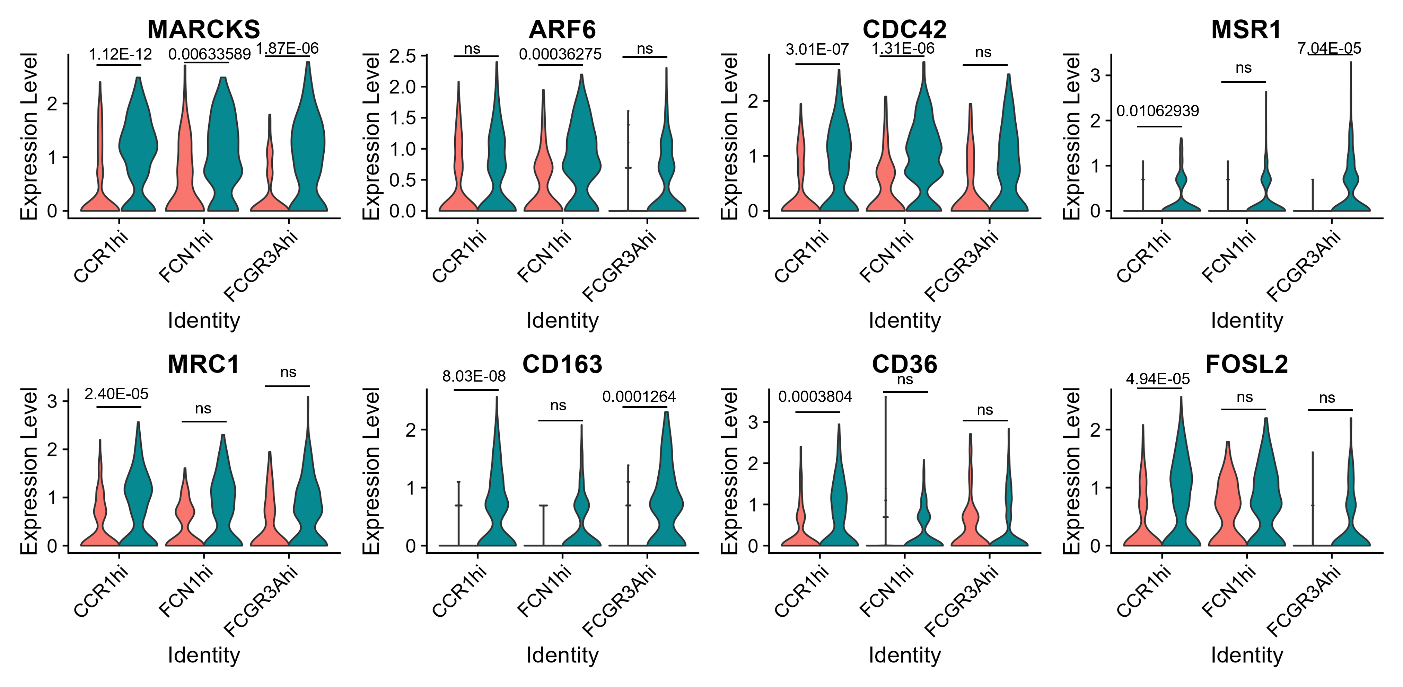

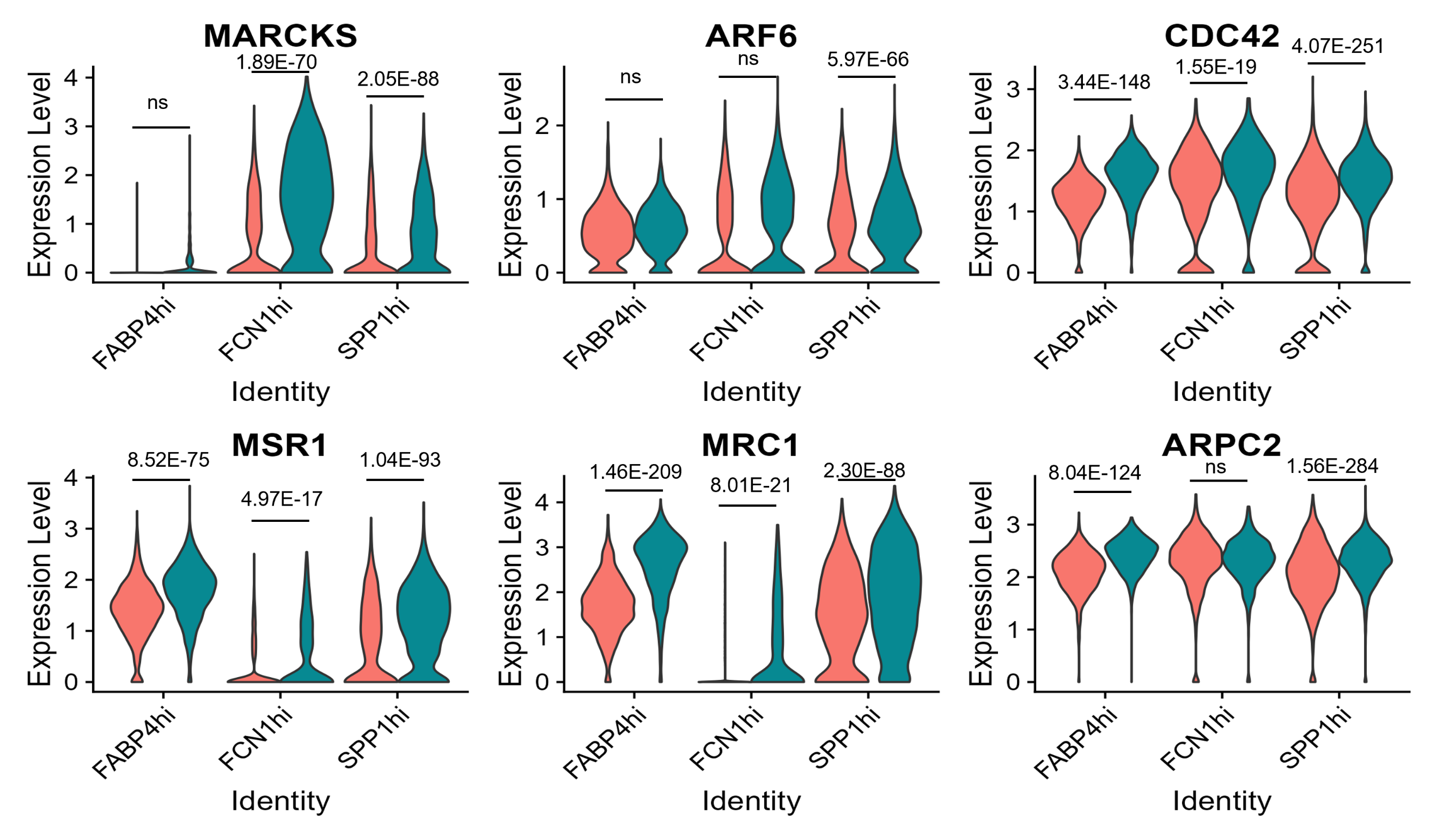


**b**

**Supplementary Fig. S2: Violin plots for genes of interest in skin and lung clusters**

**a,** Violin plots of the three main skin macrophage subclusters is shown comparing SSc patients (green) and healthy controls (red). **b,** Violin plots of the three main lung macrophage subclusters is shown comparing SSc patients (green) and healthy controls (red).

**
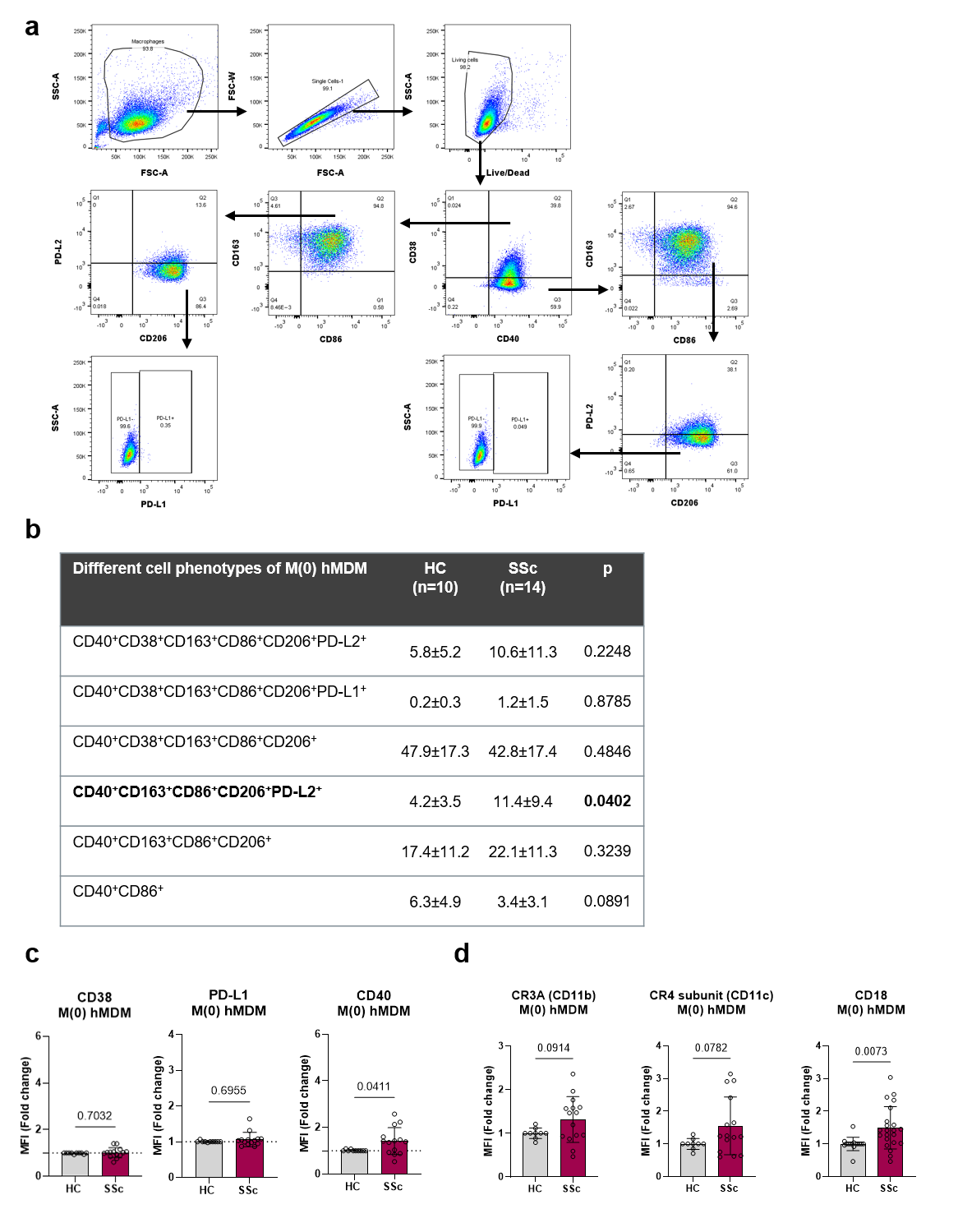
**

**Supplementary Fig. S3: Characterization of M(0) hMDM phenotype.**

**a-d**, CD14^+^ monocytes were isolated from PBMCs from healthy controls and SSc patients and differentiated into hMDM with rh M-CSF (50 ng/ml). hMDM were left unpolarized as M(0) hMDM. **a**, A representative gating scheme on M(0) hMDM from a healthy control is shown gating through CD38, CD40, CD86, CD163, CD206, PD-L2 and PD-L1 marker combinations. **b**, Percentage of different cell phenotypes of M(0) hMDM are shown. Multicolor flow cytometry analysis of the most frequent macrophage polarization populations of M(0) healthy control (n=10) and SSc patient (n=14) hMDM was performed. **c**, Polarization markers of M(0) hMDM were assessed using multicolor flow cytometry from healthy controls (n=10) and SSc patients (n=14). Quantifications of MFI (fold changes) of CD38, PD-L1, and CD40 are shown. **d**, Expression of surface CR3A (CD11b), CR4 subunit (CD11c) and CD18 of M(0) hMDM from healthy controls (n=5-15) and SSc patients (n=9-22) were assessed by flow cytometry. **b-d**, Data are shown as mean ± SD. Unpaired two-tailed parametric t-test were performed.


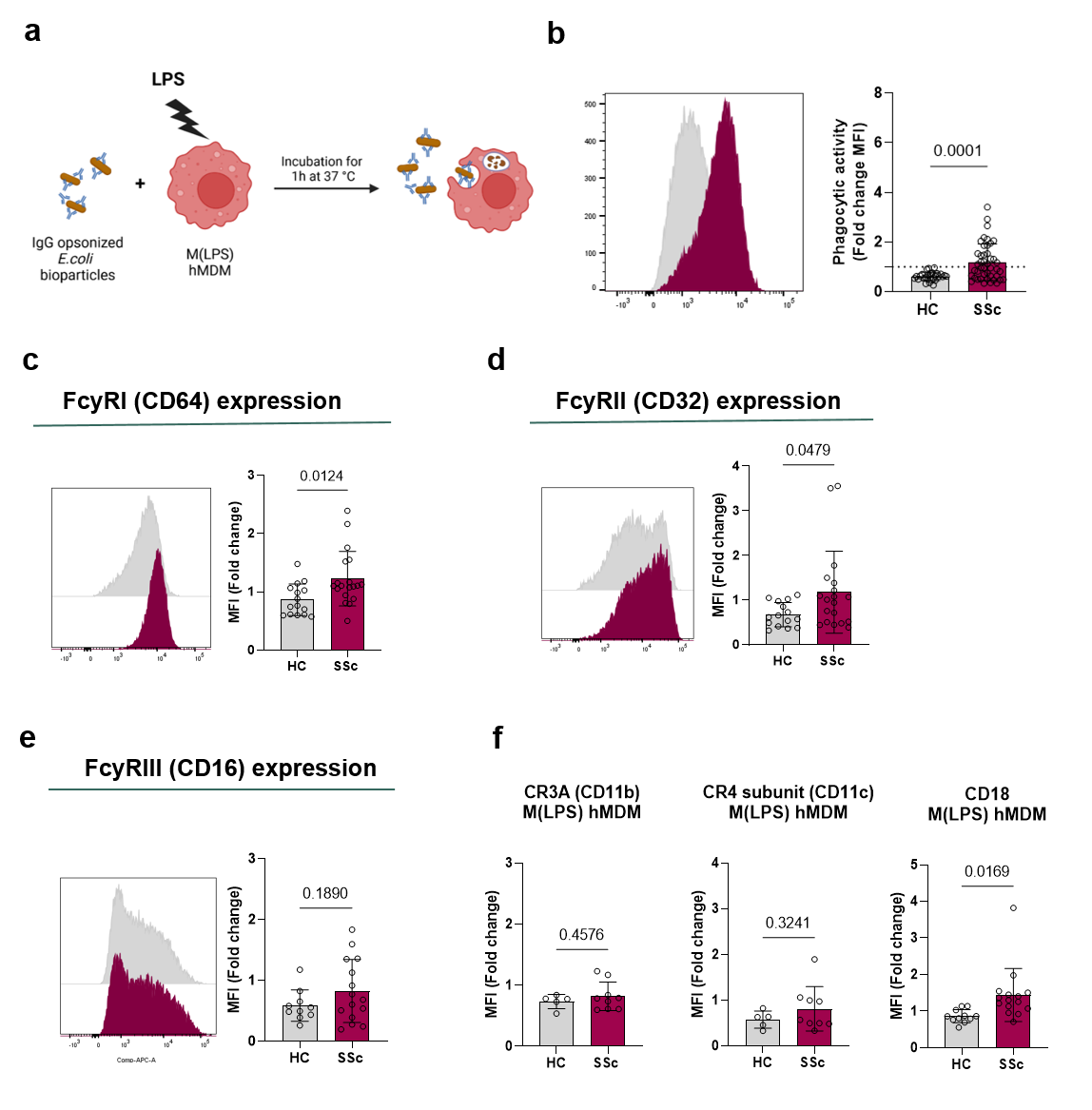


**Supplementary Fig. S4: Under pro-inflammatory conditions, SSc macrophages demonstrate heightened phagocytosis concomitant with increased FcγR expression.**

**a**, Experimental workflow of *in vitro* phagocytosis assessment in M(LPS) hMDM. M(LPS) healthy control (n=29) and SSc (n=44) hMDM were incubated for 1 hour with pHrodo Red bioparticles and the uptake was assessed by flow cytometry. **b,** A representative histogram of phagocytic activity (measured as MFI) and corresponding analysis of M(LPS) healthy control (grey histogram) and SSc (purple histogram) hMDM. **c-e**, Expression of surface FcγRI (CD64) (**c**), FcγRII (CD32) (**d**) and FcγRIII (CD16) (**e**) of M(LPS) hMDM from healthy controls (n=14-16) and SSc patients (n=15-22) were assessed by flow cytometry. **f**, Expression of surface CR3A (CD11b), CR4 subunit (CD11c) and CD18 of M(LPS) hMDM from healthy controls (n=5-15) and SSc patients (n=9-22) were assessed by flow cytometry. Data are shown as mean ± SD or median ± interquartile range. Significance was determined using unpaired non-parametric Mann-Whitney U test or unpaired two-tailed parametric t-test.


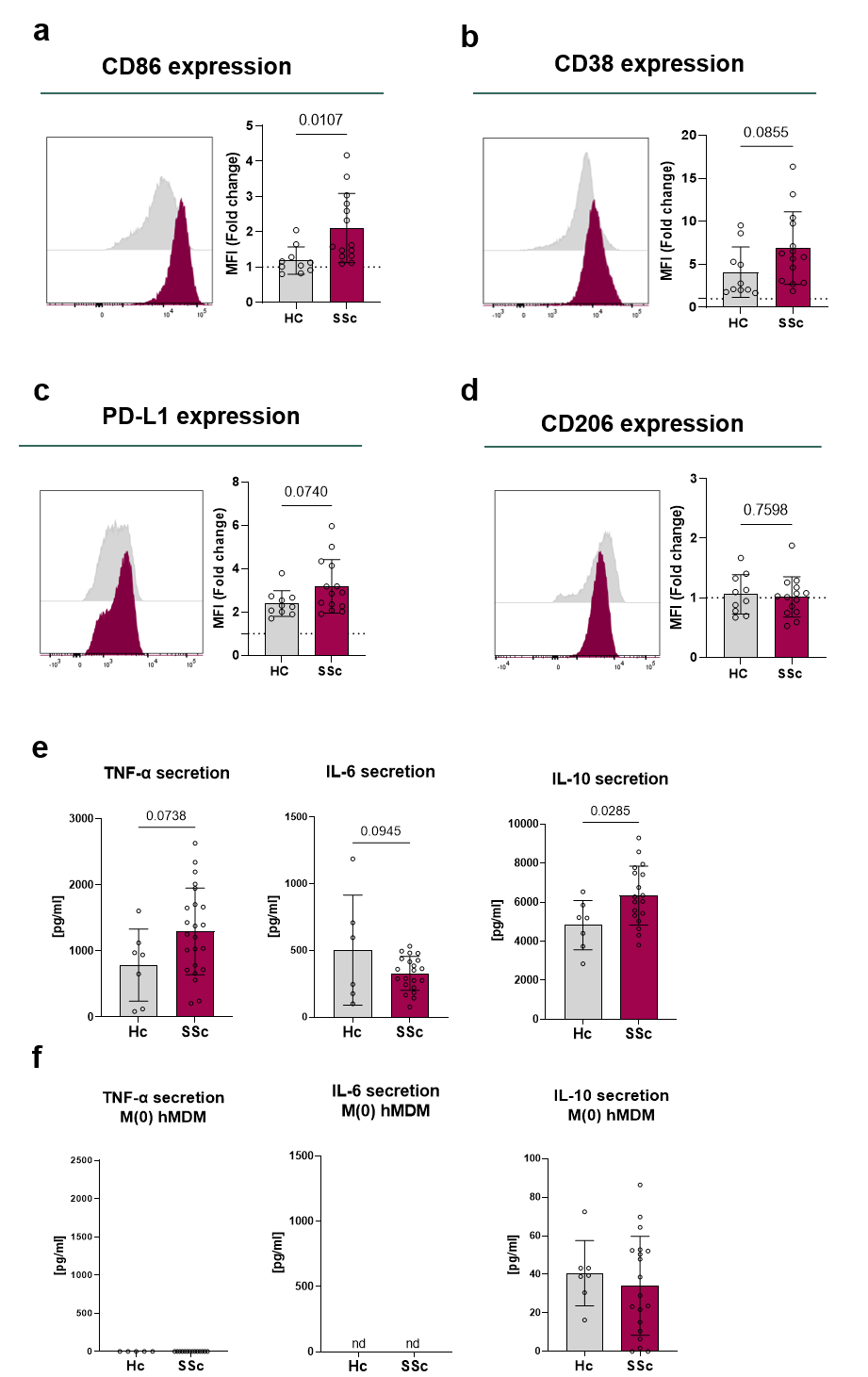


**Supplementary Fig. S5: *In vitro* phenotypical characteristics of M(LPS) hMDM.**

**a**, Polarization markers of M(LPS) hMDM were assessed using multicolor flow cytometry from healthy controls (n=10) and SSc patients (n=14). hMDM were stained with fluorescently label polarization surface markers and analyzed by flow cytometry. Representative histogram and quantification of MFI (fold changes) for CD86 (**a**), CD38 (**b**), PD-L1 (**c**), and CD206 (**d**) are shown. **e**, Cytokine concentrations of TNF-α, IL-6 and IL-10 were measured in cell culture supernatants from M(LPS) hMDM by ELISA, HC, n=7-11, SSc, n=18-24. **f**, Cytokine concentrations of TNF-α, IL-6 and IL-10 were measured in cell culture supernatants from M(0) hMDM by ELISA, HC, n=7-11, SSc, n=18-24. Data are shown as mean ± SD or median ± interquartile range. Significance was determined using unpaired non-parametric Mann-Whitney U test or unpaired two-tailed parametric t-test.


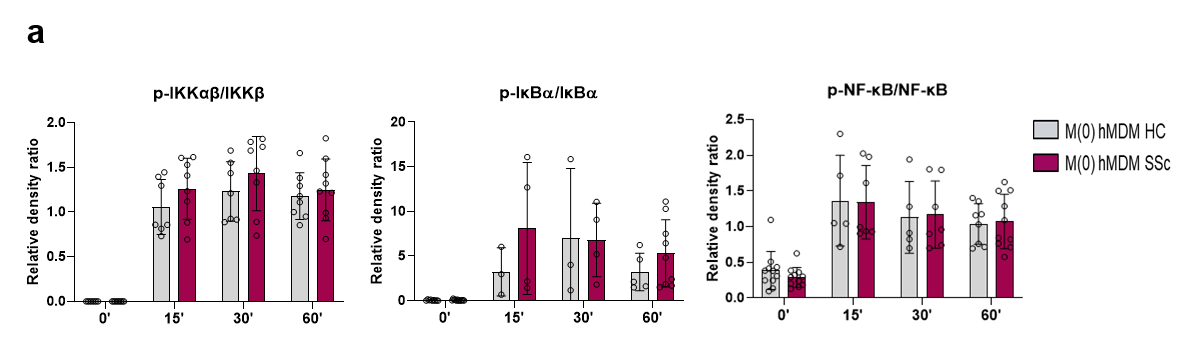


**Supplementary Fig. S6: NF-κB pathway assessment comparing healthy control and SSc patients.**

Quantification of p-IKKαβ/IKKβ, p-NF-κB/NF-κB and p-IκBa/IκBa ratios are shown comparing healthy (n=7-8) vs SSc (n=8-9) hMDM.


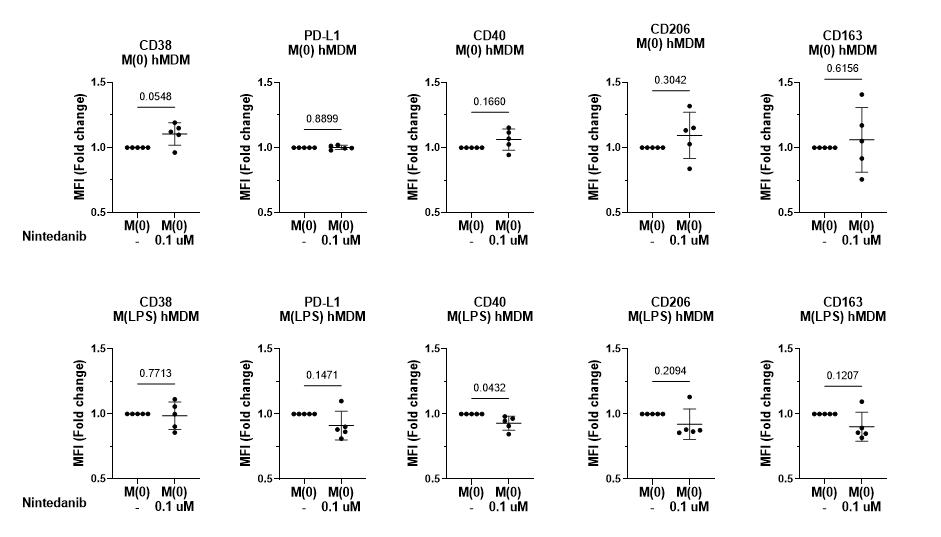


**Supplementary Fig. S7: Macrophage polarization phenotype assessment after nintedanib treatment.**

SSc hMDM were pre-treated with 0.1 μM nintedanib or were left untreated for 24 hours together with their polarization into M(0) or M(LPS). Expression of surface CD38, PD-L1, CD40, CD206 and CD163 of M(0) and M(LPS) hMDM from SSc patients (n=5) were assessed by flow cytometry. Data are shown as mean ± SD. Significance was assessed using a paired t-test.


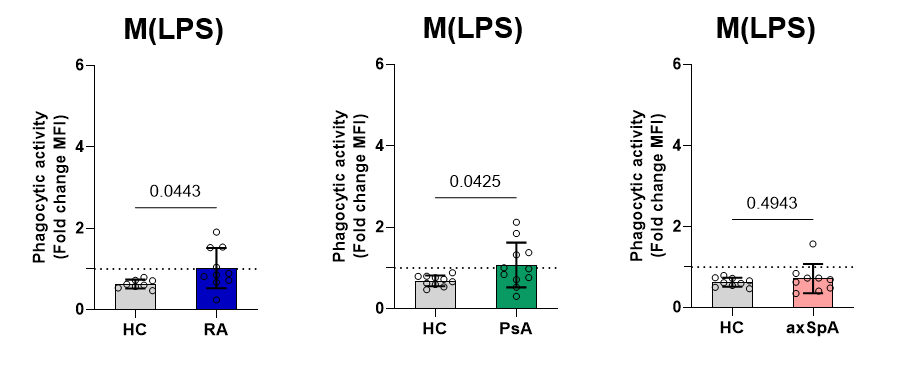


**Supplementary Fig. S8: *In vitro* phagocytosis of M(LPS) hMDM from RA, PsA, and axSpA patients under inflammatory condition.**

CD14^+^ monocytes were isolated from PBMCs from healthy controls (n=8-10), RA patients (n=10), PsA patients (n=11), and axSpA patients (n=9). CD14^+^ monocytes were then differentiated into hMDM using rh M-CSF (50 ng/ml). M(LPS) hMDM were incubated for 1 hour with pHrodo Red bioparticles and uptake was assessed by flow cytometry. Analysis of phagocytic activity (measured as MFI) of M(LPS) hMDM comparing healthy control vs RA, healthy control vs PsA, and healthy control vs axSpA patients is shown.


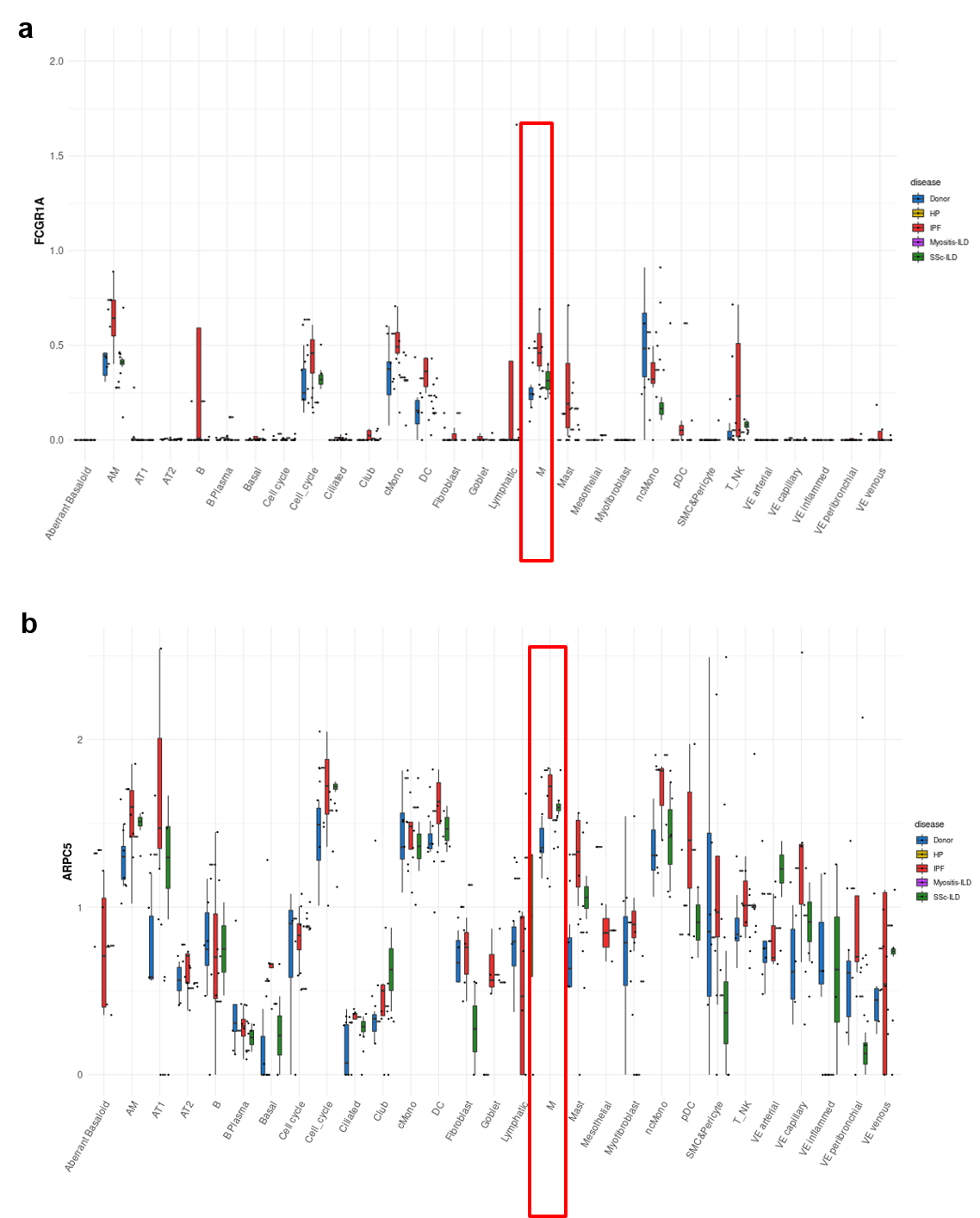
**Supplementary Fig. S9: *FCGR1A* and *ARPC5* gene expression is upregulated in IPF and SSC-ILD patients (GSE121611 dataset).**

**a-b**, Box plots of expressional differences of *FCGR1A* (**a**) or *ARPC5* (**b**) gene comparing lung cells from healthy control, hypersensitivity pneumonitis (HP), IPF, myositis-ILD and SSc-ILD patients. Highlighted in red is the comparison in the macrophage cluster. Graphs were generated from the Misharin dataset
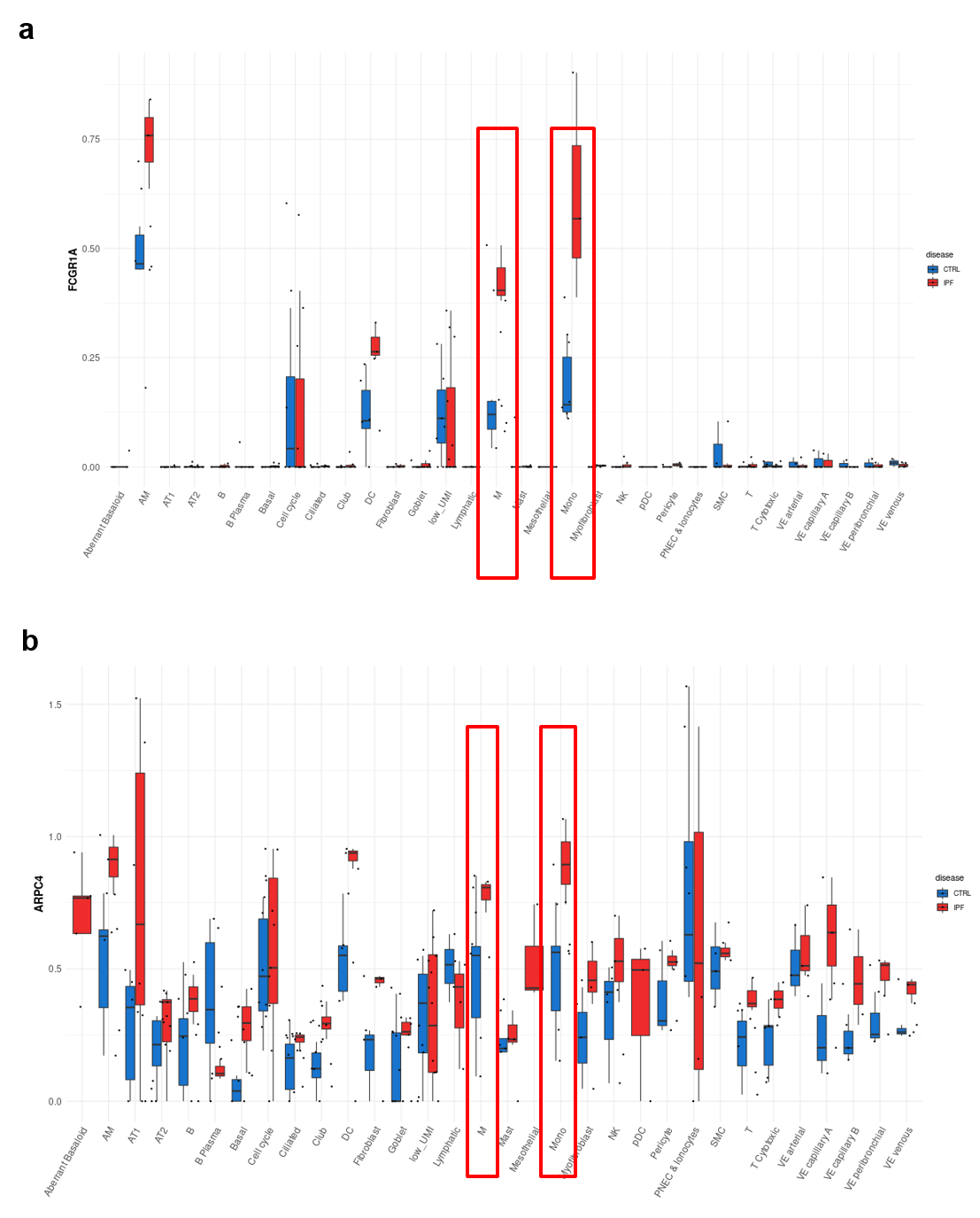
using the visualization tool available from the www.ipfcellatlas.com.

**Supplementary Fig. S10: *FCGR1A* and *ARPC4* gene expression is upregulated in IPF patients (GSE128033 dataset).**


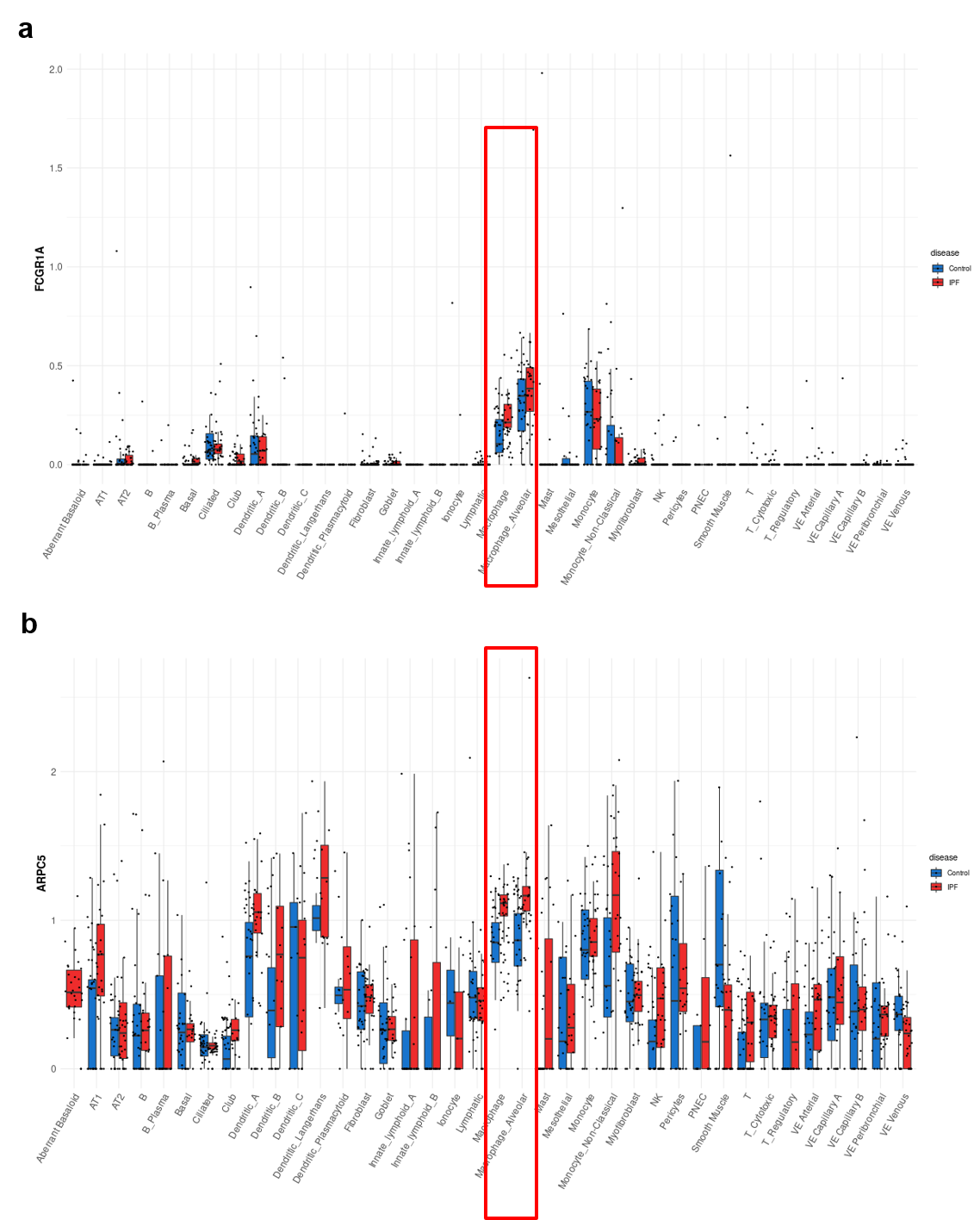
**a-b**, Box plots of expressional differences of *FCGR1A* (**a**) or *ARPC4* (**b**) gene comparing lung cells from healthy control and IPF patients. Highlighted in red are the comparison in the macrophage and monocyte cluster. Graphs were generated from the Lafyatis dataset using the visualization tool available from the www.ipfcellatlas.com.

**Supplementary Fig. S11: *FCGR1A* and *ARPC5* gene expression is upregulated in IPF patients (GSE136831 dataset).**

**a-b**, Box plots of expressional differences of *FCGR1A* (**a**) or *ARPC5* (**b**) gene comparing lung cells from healthy control and IPF patients. Highlighted in red are the comparison in the macrophage and alveolar macrophage cluster. Graphs were generated from the Rosas/Kaminski dataset using the visualization tool available from the www.ipfcellatlas.com.
